# An updated phylogeography and population dynamics of porcine circovirus 2 genotypes: are they reaching an equilibrium?

**DOI:** 10.3389/fmicb.2024.1500498

**Published:** 2024-10-29

**Authors:** Giovanni Franzo, Claudia Maria Tucciarone, Matteo Legnardi, Michele Drigo, Joaquim Segalés

**Affiliations:** ^1^Department of Animal Medicine, Production and Health, University of Padova, Legnaro, Padua, Italy; ^2^Departament de Sanitat i Anatomia Animals, Facultat de Veterinària, UAB, Barcelona, Spain; ^3^Unitat Mixta d'Investigació IRTA-UAB en Sanitat Animal, Centre de Recerca en Sanitat Animal (CReSA), Campus de la Universitat Autònoma de Barcelona (UAB), Barcelona, Spain; ^4^WOAH Collaborating Center for Research and Control of Emerging and Re-Emerging Pig Diseases in Europe (IRTA-CReSA), Barcelona, Spain

**Keywords:** porcine circovirus 2 (PCV2), genotypes, evolution, phylodynamic, phylogeography, selection

## Abstract

**Introduction:**

Porcine circovirus 2 (PCV2) emerged more than three decades ago as one of the most impactful pathogens in the swine industry. Despite being a DNA virus, one of the hallmarks of PCV2 is its high evolutionary rate, which has led to the emergence of different genotypes, each exhibiting varying degrees of evolutionary success. Current knowledge suggests the occurrence of three main waves of genotype dominance, alternating over time (i.e., PCV2a, PCV2b, and PCV2d), alongside less prevalent genotypes. However, although PCV2d is currently the most common genotype nowadays, the others continue being circulating in the pig population.

**Methods:**

The present study reconsidered the epidemiological and evolutionary patterns of PCV2 genotypes using phylodynamic analyses, benefiting from an almost 10-fold increase in ORF2 sequence availability compared to previous studies. Additionally, a phylogeographic analysis was performed to investigate viral dispersal patterns and frequency, and the selective pressures acting on the capsid protein were estimated and compared among genotypes.

**Results:**

While successive emergence of major genotypes was confirmed, this study extends previous findings by revealing subsequent prevalence fluctuations of PCV2a and PCV2b after their initial decline. This evolutionary process may represent an example of balancing selection, specifically negative frequency-dependent selection, where a genotype fitness diminishes as it becomes more common, and vice versa. Variations in genotype- or clade-specific immunity—affected by the local prevalence of viral groups—combined with the periodic introduction of strains that have independently evolved in different regions, may have led to fluctuations in the population dynamics of major genotypes over time. These fluctuations were associated with ongoing evolution and variations in the capsid amino acid profile.

**Discussion:**

These findings have profound implications for future control strategies. Although PCV2d remains the most prevalent and widespread genotype, other genotypes should not be neglected. Control strategies should thus target the entire PCV2 population, with a focus on fostering broader and more cross-protective immunity.

## 1 Introduction

Porcine circovirus 2 (PCV2) is a small, non-enveloped virus belonging to the *Circoviridae* family, within the genus *Circovirus* (Varsani et al., 2024). It is one of the smallest known viruses, characterized by a circular single-stranded DNA (ssDNA) genome that contains a limited number of open reading frames (ORFs). Among these, ORF1 is particularly important, as it codes—through alternative splicing—for Rep and Rep’ proteins, which are involved in viral genome replication. ORF2 encodes the Cap protein, the only constituent of the viral capsid, which mediates viral attachment to host cells and contains the main targets of the host immune response, including neutralizing antibodies and cell-mediated immunity epitopes. Due to its biological relevance and high genetic variability, ORF2 is the most sequenced and investigated PCV2 gene. It is commonly used for molecular epidemiology studies and for PCV2 genotyping (Franzo and Segalés, 2018). Other ORFs (ORF3-6) have been shown to be transcribed and translated, playing essential roles in apoptosis regulation and interfering with host signaling pathways at different levels (Karuppannan and Kwang, 2011; Gao et al., 2014; Lv et al., 2014; Choi et al., 2018; Li et al., 2018). Initially associated with postweaning multisystemic wasting syndrome (PMWS, currently known as PCV2 systemic disease, PCV2-SD) in the early 90s, PCV2 was thereafter involved in the pathogenesis of other clinical conditions such as PCV2 reproductive disease (PCV2-RD) and porcine dermatitis and nephropathy syndrome (PDNS). These three clinical entities (PCV2-SD, PCV2-RD, and PDNS) are collectively named porcine circovirus diseases (PCVDs) (Segalés and Sibila, 2022). These PCVDs are multifactorial, but most PCV2 infections are subclinical (PCV2 subclinical infection, PCV2-SI). Paradoxically, PCV2-SI has an economic impact that is even higher than clinical infections on farm profitability, due to decreased average daily weight gain and increased susceptibility to co-infections (Segalés and Sibila, 2022). The introduction of mass PCV2 vaccination in the first decade of the 2000s proved extremely effective, not only in preventing overt clinical signs but also in limiting losses in subclinically infected animals. Since then, vaccination has been intensively and routinely applied to the point of becoming a standard part of management on almost all intensive pig farms worldwide (Karuppannan and Opriessnig, 2017).

Despite the widespread benefits of vaccination, PCV2 has continued circulating, spreading, and evolving globally. Episodes of vaccine or vaccination apparent failure have been periodically reported, occasionally attributed to the lack of cross-protection provided by commercial vaccines based on the capsid protein of PCV2a genotype against other genotypes (Xiao et al., 2012; Seo et al., 2014). Nowadays there is a broad consensus in the literature regarding the efficacy of current vaccines; the so-called vaccination failures are most likely due to improper vaccination management since evidence suggests that variable but significant cross-protection among strains and genotypes does exist (Opriessnig et al., 2017, 2020a,b; Franzo and Segalés, 2020). This variability could nevertheless have played a role in shaping the epidemiology of PCV2, influencing the virus ongoing evolution and persistence across different regions (Franzo et al., 2016b; Franzo and Segalés, 2020). Like other ssDNA viruses, PCV2 exhibits a high evolutionary potential due to both high mutation and recombination rates, leading to the emergence of several genetic variants. These variants are classified into genotypes based on a combination of genetic distance, phylogenetic analysis, and comparison with reference strains (Franzo and Segalés, 2018). Currently, nine genotypes have been proposed, varying in frequency, geographical distribution, and potentially biological characteristics (Franzo and Segalés, 2018; Wang et al., 2020). However, differences in virulence and immunogenicity among these genotypes are still debated, and within-genotype heterogeneity also occurs.

Among PCV2 genotypes, only three (PCV2a, PCV2b, and PCV2d) are considered major genotypes due to their high prevalence, worldwide distribution, and persistent circulation over the years (Franzo et al., 2016a; Franzo and Segalés, 2018). The first genotype to be identified was PCV2a, which was displaced as the dominant one by PCV2b around 2000–2003 (first genotype shift) and finally by PCV2d after 2010–2014 (second genotype shift). The rise of PCV2d, which appears to be progressively replacing other genotypes, has been linked, at least to some extent, to the widespread application of PCV2a-based vaccines (Franzo et al., 2016a,b). These vaccines may confer differential immunity against various genotypes, which, although effective from a clinical perspective, might provide an evolutionary advantage to certain strains. This hypothesis is supported by changes in relative genotype prevalence following the introduction of vaccination, the presence of differential selective pressures, and within-genotype clade selection (Shen et al., 2012; Reiner et al., 2015; Franzo et al., 2016a; Franzo and Segalés, 2020). Moreover, experimental studies have provided evidence of more effective protection against homologous strains compared to heterologous challenges, and vaccination performed combining strains belonging to different genotypes seems to confer better protection (Bandrick et al., 2022).

However, despite the described trends, all major genotypes are still consistently reported, albeit with varying frequencies, across the globe. As a result of the extensive short- and long-distance dispersal of PCV2 strains, the co-circulation and competition of different strains and genotypes—each potentially exhibiting different biological and immunological features—have likely contributed to shaping the current epidemiological landscape in a less predictable manner than what might be expected in the presence of vaccine-derived immunity alone. Nonetheless, existing studies reconstructing the dynamics of PCV2 genotypes over time on a worldwide scale are almost 10 years old.

Therefore, the purpose of the present study was to investigate the population dynamics and evolution of PCV2 by taking advantage of the ever-increasing availability of sequences collected from a broader range of countries across different continents. This approach aimed to provide an updated view of the current PCV2 phylodynamic scenario.

## 2 Materials and methods

### 2.1 Datasets preparation

All complete or nearly complete PCV2 ORF2 sequences for which collection date and country of origin were available were downloaded from GenBank. Sequences containing premature stop codons and frameshift mutations, identified using dedicated Python scripts, were removed from the dataset. A preliminary alignment was performed at the amino acid level using the MAFFT method (Standley, 2013) implemented in TranslatorX (Abascal et al., 2010), then sequences were back-translated to DNA. Sequences with regions of poor-quality alignment were also removed from the database through visual inspection. The selected sequences were then aligned with a set of reference sequences suggested by Franzo and Segalés (2018) using the same program. A maximum likelihood (ML) phylogenetic tree was reconstructed with IQ-Tree (Nguyen et al., 2015), selecting the substitution model with the lowest Akaike information criterion (AIC) calculated by the software. The robustness of the inferred clades was assessed by performing 10,000 ultrafast bootstrap replicates. Sequences were classified into genotypes based on clustering with reference sequences, and independent datasets were generated accordingly. The absence of a significant within-dataset recombination signal was assessed using GARD (Kosakovsky Pond et al., 2006).

Since the main aim of the study was to focus on the current dynamics of the most epidemiologically relevant clades rather than to reconstruct the ancient history and origin of genotypes, only strains strongly clustering within recognized genotypes were considered. Strains that were part of minor clades, located on intermediate branches among genotypes, or with limited bootstrap support were not included. Although seemly arbitrary, these inclusion criteria were applied with the understanding that emphasizing groups of strains with limited or negligible epidemiological relevance—potential epidemiological dead ends—could come at the risk of genotypes misclassification or inclusion of potential recombinant strains, whose identification is often challenging and could negatively impact the accuracy of parameter estimates.

For this reason, PCV2c and the so-called PCV2d-1 clade were not considered - although of historical importance - due to the limited sequence availability and current limited relevance. PCV2i was also not explicitly considered since it closely clustered with PCV2a and some PCV2i strains were initially classified within PCV2a. However, to avoid classification assumptions affecting results reliability, analyses were repeated considering PVC2i as an independent genotype.

Sampling and sequencing bias due to different resource availability or attitudes to perform molecular epidemiology investigations is known to occur (Hall et al., 2016; Layan et al., 2023). To obtain more balanced datasets and reduce the computational burden, each genotype-specific dataset was down-sampled by randomly selecting up to four sequences for each country-year pair (Country-year dataset). Additionally, another random dataset of 500 sequences was generated (Balanced dataset) for each genotype by randomly selecting sequences according to a probability function that accounted for the time-country composition of the original dataset. No down-sampling was performed on minor genotypes due to their limited number.

### 2.2 Phylodynamic and phylogeographic analysis

The selected datasets were analyzed to estimate several population parameters, including the time to the most recent common ancestor (tMRCA), evolutionary rate, and viral population dynamics, using the Bayesian serial coalescent approach implemented in BEAST 1.10 (Suchard et al., 2018). For each dataset, the nucleotide substitution model was chosen based on the Bayesian Information Criterion (BIC) score calculated using JmodelTest2 (Darriba et al., 2012). The lognormal relaxed molecular clock (Drummond et al., 2006) model was preferred to the strict molecular clock by calculating the marginal likelihood estimation through path-sampling and stepping-stone methods, as recommended by Baele et al. (2013). The non-parametric Bayesian Skygrid model was used to reconstruct viral population changes over time, focusing on relative genetic diversity (effective population size multiplied by generation time; Ne x *τ*) (Hill and Baele, 2019).

For the genotype-specific datasets, a discrete state phylogeographic analysis was also conducted, following the method described by Lemey et al. (2009). This analysis implemented an asymmetric migration model with Bayesian stochastic search variable selection (BSSVS) to identify the most parsimonious representation of the viral spread and calculate a Bayesian Factor (BF) that indicates the statistical significance of inferred migration paths between geographic regions. Due to the sparse nature of sequence-country combinations and likely gaps in sampling from several countries, the datasets were also analyzed by aggregating countries into macro-geographical areas based on spatial proximity and geopolitical factors (e.g., Africa, Asia, Europe, North America, Oceania, and South America).

Two independent runs of 100 million generations were performed. The log and tree files were merged using LogCombiner after discarding 20% as burn-in. The results were analyzed using Tracer 1.7 and were considered valid only if the estimated sample size (ESS) exceeded 200 and the convergence and mixing were adequate. Parameter estimates were summarized using mean values and 95% highest posterior density (HPD) intervals. Maximum clade credibility (MCC) trees were constructed and annotated using TreeAnnotator (part of the BEAST package). SpreaD3 (Bielejec et al., 2016) was utilized to calculate the BF associated with each migration route. All non-zero transition rates between countries were deemed significant if the calculated BF exceeded 5. Additional summary statistics and graphical outputs were generated using custom R and Python scripts.

To estimate the relevance of major genotypes in different geographic areas over time, the proportion of lineages circulating in each region was reconstructed by analyzing the MCC tree annotated with ancestral state reconstruction. The number of branches assigned to a specific area, based on the highest posterior probability, was counted at different time points. Specifically, 50 equally spaced time intervals were defined from the most recently collected sample to the tMRCA. A smoothing function was applied to depict a continuous trend over time and to mitigate local, inconsistent trends that could arise due to estimation uncertainty.

### 2.3 Selective pressure analysis

The analysis of pervasive selective pressures on the Cap protein across major genotypes was conducted using FUBAR (Murrell et al., 2013), while MEME was utilized to identify sites under episodic diversifying selection (Murrell et al., 2012). A posterior probability (PP) > 0.9 was deemed significant for FUBAR, and a *p*-value <0.05 was used for MEME. To assess differences in selective pressure strength among genotypes, contrast-FEL was employed (Kosakovsky Pond et al., 2021). A false discovery rate (FDR)-corrected *q*-value of 0.2 was set as the threshold for detecting overall site-specific differences in selective pressure, with a *p* < 0.05 used for comparisons between genotype pairs. All analyses were performed using HyPhy (Kosakovsky Pond et al., 2005) or the Datamonkey web server (Weaver et al., 2018). The identified features of interest were mapped onto the capsid quaternary structure, for which the 3R0R assembly was downloaded from PDB and visualized and edited in Chimera (Pettersen et al., 2004).

## 3 Results

### 3.1 Sequence dataset

A total of 8,283 sequences were included in the final dataset (Supplementary Table S1). A summary of genotype-specific metadata of the included strains is provided in Table 1. After database refinement, no evidence of recombination was detected in each dataset.

**Table 1:** Summary of sequences included in the study.

Genotype	N	Years	Countries	tMRCA	Evolutionary rate
PCV-2a	1,091	1996–2024	Belgium(3), Brazil(4), Canada(10), CapeVerde(2), Chile(10), China(413), Colombia(2), Croatia(2), Democratic Republic of the Congo(3), Ethiopia(2), France(6), Germany(16), India(3), Italy(18), Japan(40), Malaysia(2), Mexico(4), Mongolia(1), Mozambique(1), Netherlands(3), Nigeria(8), Poland(6), Portugal(6), Romania(5), Russia(1), Senegal(2), Serbia(3), Slovakia(5), South Korea(40), Spain(3), Switzerland(1), Taiwan(14), Tanzania(2), Thailand(7), USA(433), Ukraine(7), United Kingdom(2), Vietnam(1)	1940.381 [95HPD:1919.225–1958.202]	9.154×10^−4^ [95HPD:7.955×10^−4^-1.046×10^−3^]
PCV-2b	2,670	1999–2024	Argentina(14), Australia(2), Belarus(1), Belgium(2), Brazil(64), Canada(9), Chile(7), China(1184), Croatia(12), Cuba(1), Denmark(9), Ethiopia(1), France(29), Germany(30), India(8), Indonesia(1), Italy(147), Japan(37), Lithuania(6), Malaysia(5), Mexico(7), Mongolia(1), Mozambique(1), Namibia(9), Netherlands(1), Nigeria(2), Paraguay(1), Poland(9), Portugal(18), Romania(36), Russia(6), SaintKittsandNevis(16), Serbia(6), Slovakia(36), SouthAfrica(11), SouthKorea(101), Spain(2), Sweden(1), Switzerland(4), Taiwan(28), Thailand(55), USA(579), Ukraine(31), UnitedKingdom(75), VietNam(65)	1993.824 [95HPD:1985.97–1999.699]	7.553×10^−4^ [95HPD:4.267×10^−4^-1.141×10^−3^]
PCV-2d	4,303	2003–2024	Argentina(13), Austria(1), Belgium(6), Bhutan(2), Brazil(22), Burkina Faso(1), Cambodia(5), Canada(13), Chile(8), China(2077), Colombia(77), Denmark(16), Dominican Republic(17), Ethiopia(4), France(56), Germany(40), Hungary(6), India(55), Indonesia(1), Italy(238), Japan(91), Malaysia(77), Mexico(58), Mozambique(4), Netherlands(6), Nigeria(25), Poland(17), Portugal(1), Romania(4), Russia(30), Serbia(2), South Africa(3), South Korea(184), Spain(24), Taiwan(241), Tanzania(16), Thailand(137), USA(555), Ukraine(5), United Kingdom(7), Uruguay(5), Vietnam(153)	1985.702 [95HPD:1966.41–1995.375]	6.837×10^−4^ [95HPD:5.009×10^-4-^8.832×10^−4^]
PCV-2e	42	2006–2022	China(8), Italy(9), Mexico(1), South Korea(2), Taiwan(1), USA(21)	1980.609 [95HPD:1958.747–1994.778]	4.165×10^−4^ [95HPD:2.325×10^−4^-7.618×10^-4^]
PCV-2f	41	1999–2018	China(11), Croatia(2), India(23), Indonesia(4), Ukraine(1)	1919.636 [95HPD:1799.726–1982.876]	6.053×10^−4^ [95HPD:1.920×10^−4^-1.649×10^−3^]
PCV-2 g	34	1999–2019	China(4), India(11), Mongolia(1), Romania(10), South Korea(5), Switzerland(1), Ukraine(1), Vietnam(1)	1971.456 [95HPD:1941.652–1989.989]	1.530×10^−3^ [95HPD:8.623×10^−4^-2.664×10^−3^]
PCV-2 h	102	2004–2021	China(15), India(7), Indonesia(1), Japan(1), Romania(2), South Korea(1), Thailand(18), Vietnam(57)	1979.823 [95HPD:1942.078–1997.499]	6.253×10^−4^ [95HPD:3.846×10^−4^-9.463×10^−4^]

Sequence numbers, collection date ranges, countries (with the number of sequences reported for each county in brackets), tMRCA, and evolutionary rates are reported for each genotype.

### 3.2 Phylodynamic analysis

**Figure 1:**
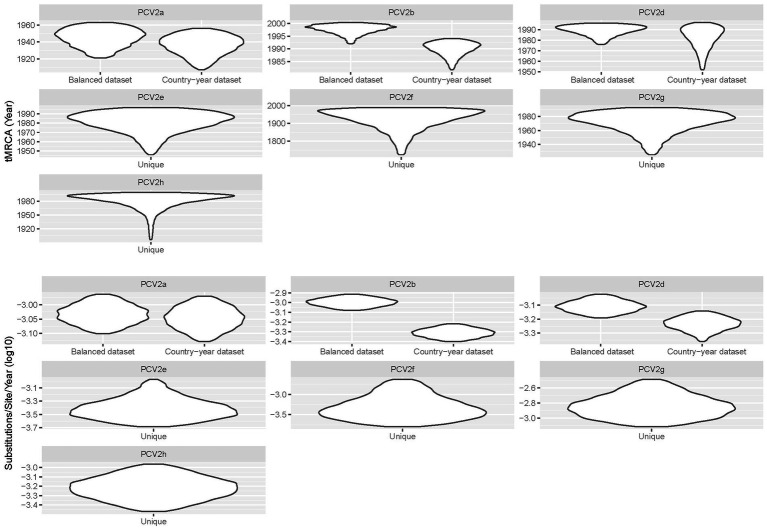
Violin plot depicting the posterior probability estimation of tMRCA and evolutionary rate calculated for different genotypes. For major genotypes, the results obtained from the analysis of the Country-year and Balanced datasets are also reported.

The tMRCA ranged from the beginning of the XX century to the 90s, depending on the considered genotype (Table 1). Different genotypes showed substantially consistent evolutionary rates, in the order of 10^−4^–10^−3^ substitution/site/year. The comparison of tMRCA and evolutionary rate calculated on the Country−year dataset with the Balanced dataset provided concordant results (Figure 1). Only PCV2b showed an apparently higher evolutionary rate and thus a more recent origin with the Balanced dataset, although a certain 95HPD overlap occurred.

**Figure 2:**
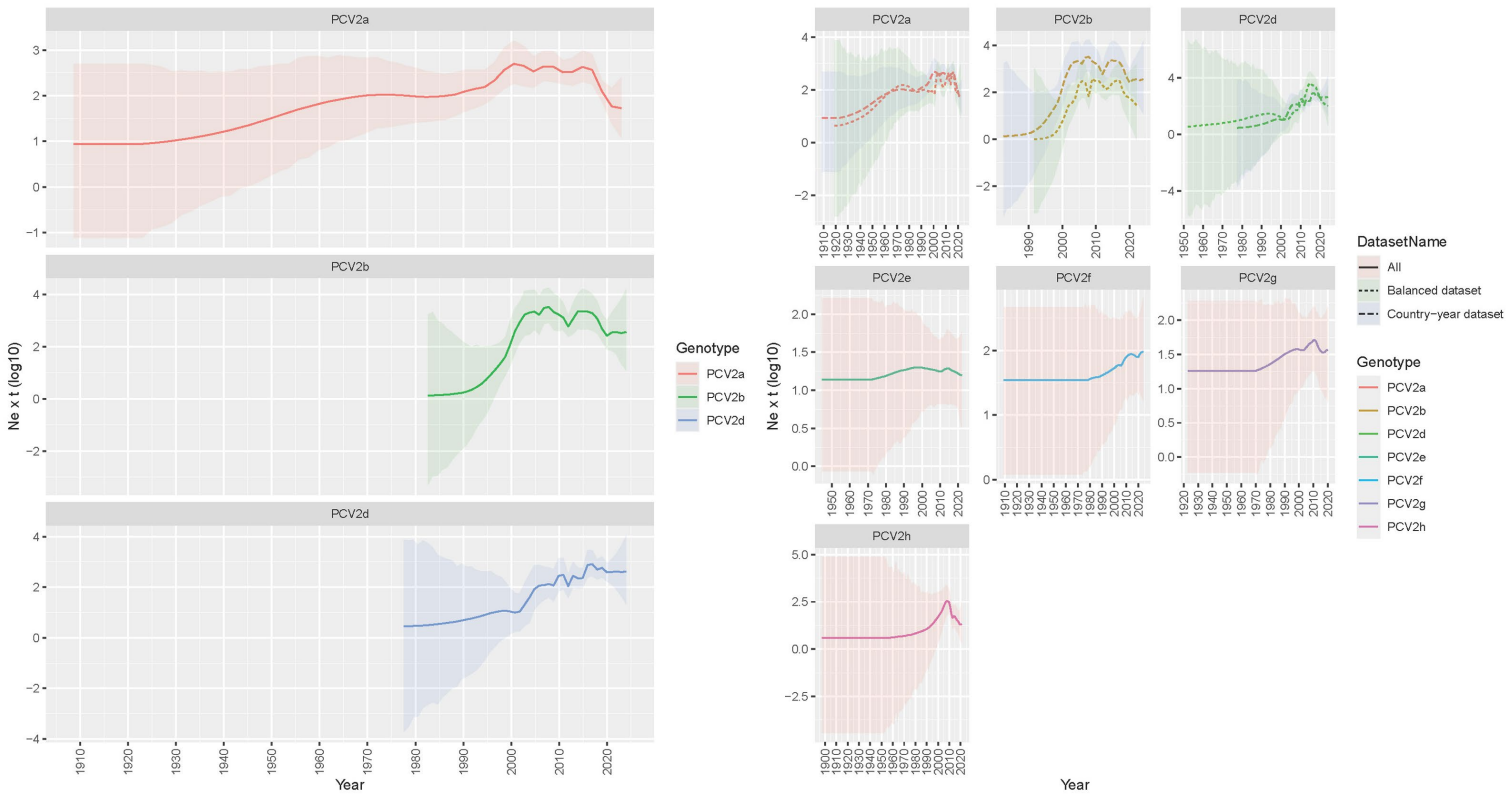
PCV2 population dynamics plot: the left graph illustrates the mean relative genetic diversity (Ne x t) of major PCV2 genotypes, represented by a solid line, while the shaded area indicates the 95% highest posterior density (HPD) interval. In the right panel, the same representation is applied to depict all PCV2 genotypes studied. Where applicable, comparisons between the Country-year and Balanced datasets are shown using different line styles.

The reconstruction of viral population dynamics based on the Country-year dataset estimated the origin of PCV2a around the beginning of the 20th century. The viral population exhibited a gradual increase following the second half of the 20th century, reaching a peak around the year 2000. This was followed by an apparent decline, with subsequent secondary fluctuations occurring approximately in 2010 and 2015, ultimately leading to a relatively stable population until a final decrease (Figure 2). The origin of PCV2b, estimated to have occurred in the 1980s, was associated with a sharper increase in relative genetic diversity, peaking around 2010. Following this peak, there was a trend toward stabilization and a progressive decline, albeit with several fluctuations. Specifically, a temporary decrease after 2005 was followed by a resurgence around 2015, culminating in a final decline and stabilization by the end of the study period (Figure 2). PCV2d, which shares a similar time to the most recent common ancestor (tMRCA) with PCV2b, exhibited a comparable but delayed pattern, with a progressive and slow increase until 2000, after which a sharper increase occurred, peaking around 2015 and stabilizing thereafter. In all instances, the Balanced dataset provided comparable patterns; however, the viral population increases for both PCV2a and PCV2b were estimated to have a delay of approximately 5 years compared to the Country-year dataset. Additionally, the fluctuations in the Balanced dataset were slightly more pronounced and less smoothed than those observed in the Country-year dataset (Figure 2). The so-called minor genotypes displayed a common trend, characterized by an essentially constant population with a tendency toward a slow, consistent increase, particularly during the period from 2000 to 2015. Notably, PCV2g and PCV2h exhibited a decline in recent years, while PCV2e and PCV2f demonstrated a more stable situation.

**Figure 3:**
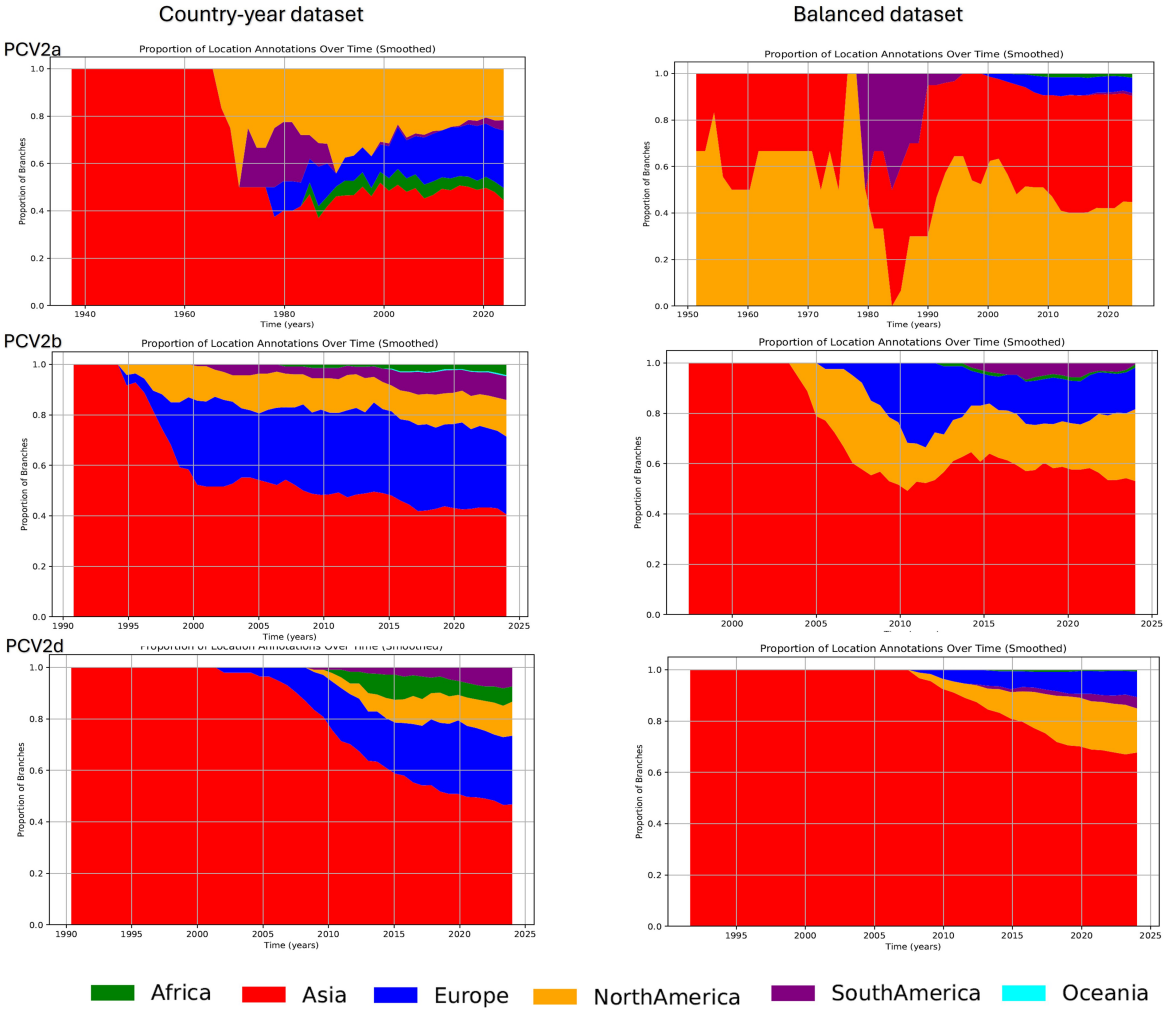
Plot depicting the proportion of lineages over time: this figure illustrates the proportion of lineages over time for PCV2a, PCV2b, and PCV2d, based on the analysis of maximum clade credibility (MCC) trees annotated with the reconstruction of ancestral states (i.e., continents). Different geographic areas are represented by distinct color coding. The results derived from the Country-year dataset are shown on the left panel, while those from the Balanced dataset are displayed on the right panel.

The reconstruction of lineages over time, despite some differences in the relative proportions across continents, depicted an overall common trend characterized by the progressive geographic expansion of the major genotypes. Initially, following the inferred tMRCA, an exclusive circulation in Asia was inferred. However, this was followed by a gradual expansion and a more balanced global distribution of PCV genotypes, with Europe and North America emerging as the primary locations harboring most lineages after Asia. A similar trend was observed when analyzing the balanced dataset. The most notable difference occurred with PCV2a, where the Country-year dataset suggested a predominantly Asian circulation until the 1980s, whereas the Balanced dataset estimated also the presence of this genotype in North America during the same period. Beyond this point, the results were consistent across datasets, although the African and European lineages, despite present, were less relevant in the balanced dataset. In the reconstruction of the PCV2b and PCV2d genotypes over time, although a similar trend was observed regardless of the dataset used, the Balanced dataset inferred a higher percentage of lineages circulating in Asia, while a higher presence in Europe instead of North America was reported in the Country-year dataset (Figure 3). In addition, the number of estimated African lineages was lower in the Balanced dataset compared to the Country-year.

The evaluation of location-annotated MCC trees revealed the presence of distinct geographical clustering. However, clades from different continents were interspersed throughout the phylogenetic tree, indicating frequent strain exchanges among different regions, as confirmed by BF-supported migration rates, followed by local independent evolution (Supplementary Figures S1, S2). Additionally, single sequences or small clades were observed, suggesting the occurrence of epidemiological dead ends. The mixing of strains from different origins and the detection of minor clades were more commonly observed in the Country-year dataset.

### 3.3 Selective pressure analysis

**Figure 4:**
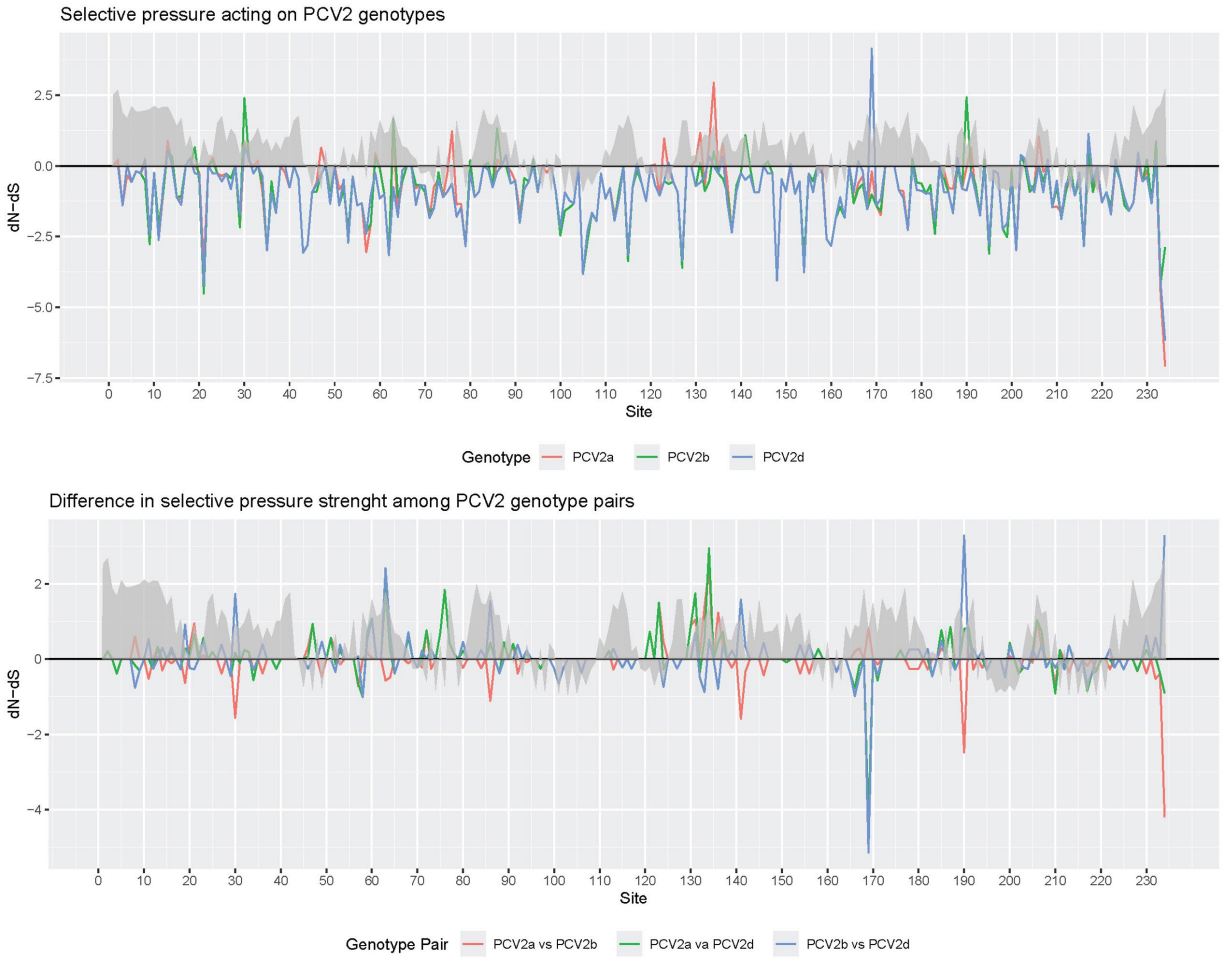
Plot depicting the dN-dS ratios of individual genotypes (upper panel) and differences among genotypes (lower panel) at various capsid positions: this figure presents the dN-dS ratios for individual PCV2 major genotypes (color-coded) in the upper panel and the differences among these genotypes in the lower panel (color-coded), focusing on different capsid positions. The plot also overlays the relative surface accessibility (RSA), calculated using the NetSurfP-2.0 server, represented by a gray area. The surface accessibility threshold is set at 25%, as predefined by the software.

**Figure 5:**
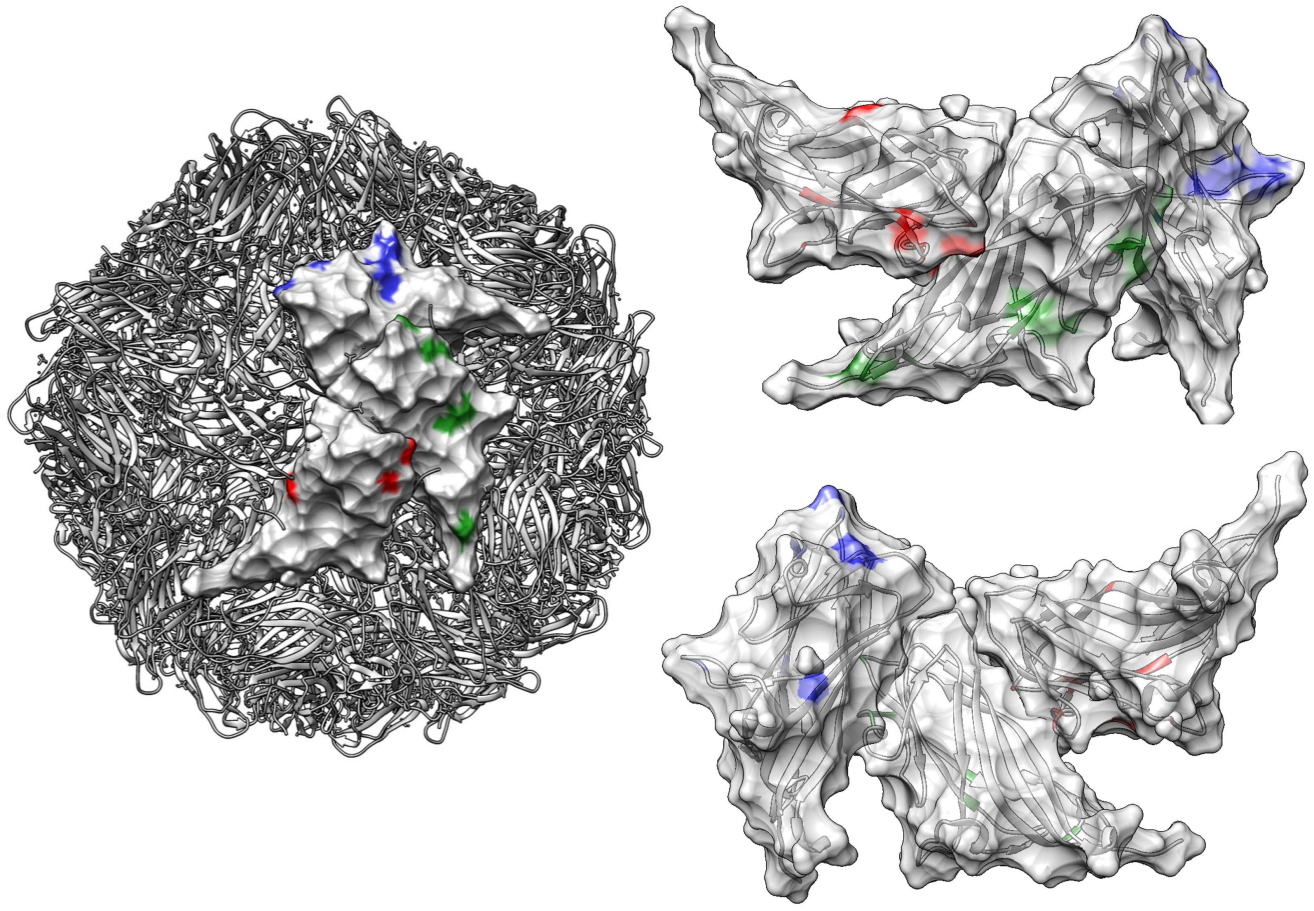
Quaternary structure of PCV2: this figure illustrates the quaternary structure of PCV2, highlighting sites under significant episodic diversifying selection as detected by MEME. These sites are color-coded in red, green, and blue for PCV2a, PCV2b, and PCV2d, respectively, on the viral surface of different capsid proteins. In the right panel, the same positions are highlighted on both the external (upper image) and internal (lower image) surfaces of three contiguous capsids. The ribbon structure, also color-coded to correspond with the selection sites, is displayed transparently in the background.

The analysis of sites under pervasive diversifying selection detected positions 47, 63, 134, 191, and 206 for PCV2a, 80, 134, and 190 for PCV2b and 134 and 169 for PCV2d. Episodic diversifying selection was reported at positions 47, 59, 63, 130, 134, 137, 169, 190, and 191 for PCV2a, 2, 3, 30, 59, 68, 80, 88, 130, 131, 134, 190, and 228 for PCV2b and 12, 59, 68, 95, 113, 134, 169, and 234 for PCV2d (Figures 4, 5).

The analysis of positions under diversifying selection shared among genotypes highlighted a turnover of the prevalent amino acid among genotypes in specific positions over time, i.e., the increase in the prevalence of one amino acid in one genotype was sometimes mirrored by a decrease in its frequency in at least one of the other groups (Supplementary Figure S3).

**Figure 6:**
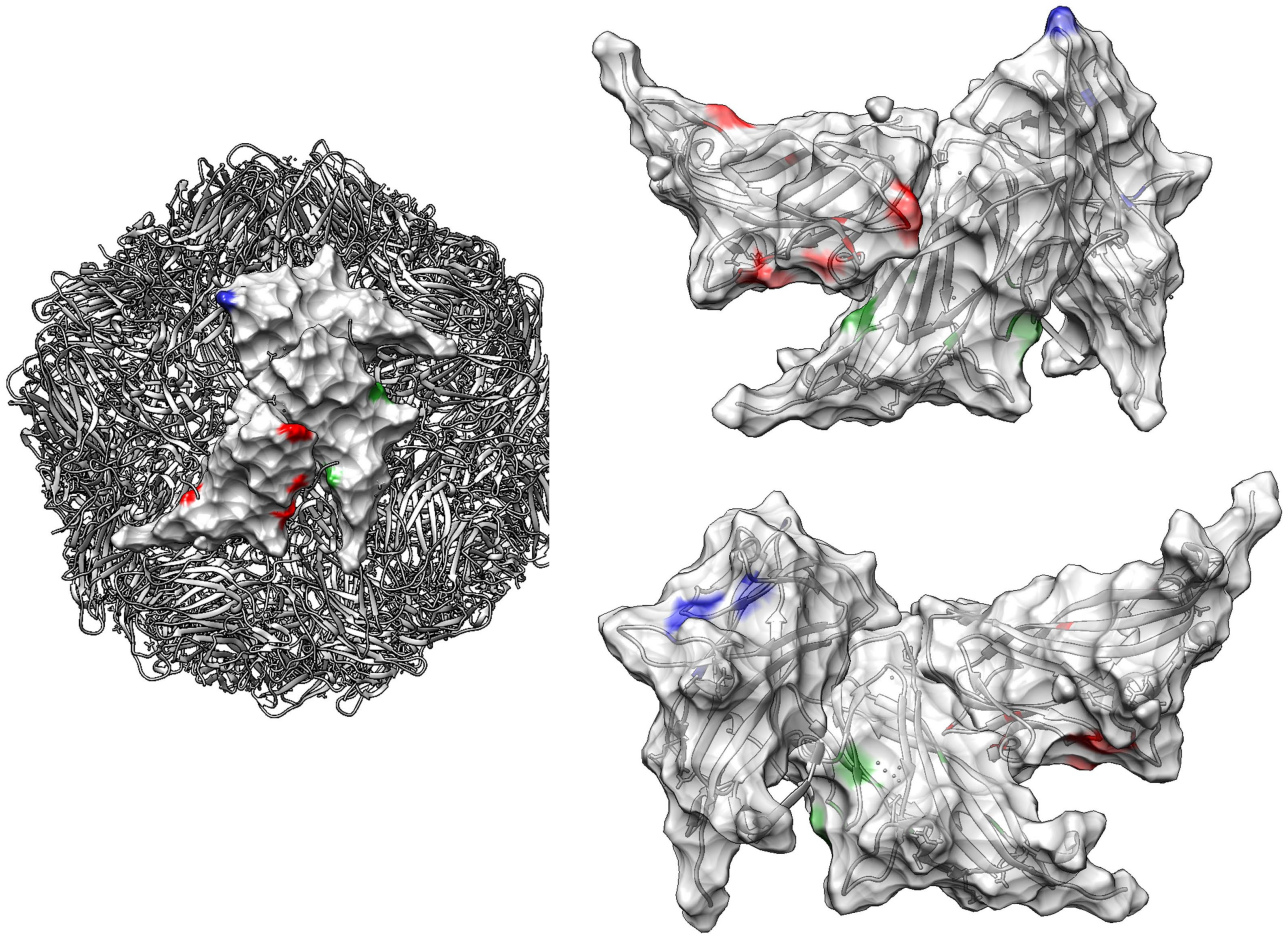
Quaternary structure of PCV2: this figure illustrates the quaternary structure of PCV2, highlighting sites under significantly different selective pressure among genotypes as detected by contrast-FEL. These sites are color-coded in red, green, and blue for PCV2a vs. PCV2b, PCV2a vs. PCV2b, and PCV2b vs. PCV2d, respectively, on the viral surface of different capsid proteins. In the right panel, the same positions are highlighted on both the external (upper image) and internal (lower image) surfaces of three contiguous capsids. The ribbon structure, also color-coded to correspond with the selection sites, is displayed transparently in the background.

Finally, the comparison of selective pressures strength among genotype pairs, estimated through contrast-FEL, identified a different selective pressure between PCV2a and PCV2b at amino acid positions 76, 123, 141, 190, 206, and 210, between PCV2a and PCV2d at positions 58, 63, 76, 123, 131, 134, 169, and 210, and at positions 58, 63, 141, 169, and 190 between PCV2b and PCV2d (Figures 4, 6).

Overall, PCV2a showed a higher variability in amino acid profile and thus diversification tendency in the considered position compared to PCV2b and PCV2d. When PCV2b and PCV2d were compared, a higher variability featured PCV2b with the main exception of position 169, where a progressive replacement of R → G over years featured PCV2d (Supplementary Figure S4).

## 4 Discussion

Porcine circovirus 2 is often considered a relatively simple virus under many aspects; however, its genetic background and evolutionary trajectories have proven to be complex and continue to challenge our understanding (Finsterbusch and Mankertz, 2009; Opriessnig et al., 2020a). The theory of genotype shift has been extensively studied and generally confirmed by molecular epidemiology surveys (Dupont et al., 2008; Cortey et al., 2011; Franzo et al., 2016a). The mechanism underlying genotype replacement has been attributed to the competitive advantage of certain genetic groups or clades, likely due to differences in virulence, tropism, replication rate or cross-protection. The widespread use of PCV2a-based vaccines is frequently cited as a significant factor contributing to the development of differential fitness among genotypes, which has been particularly detrimental to PCV2a (Kang et al., 2019). However, despite almost two decades have passed since the widespread application of commercial vaccines and the occurrence of genotype shifts, both PCV2a and PCV2b continue to be detected, albeit at lower and variable frequencies (Supplementary Figure S5). In this context, the present study not only confirms the ongoing upward trend of PCV2d but also reconstructs the occurrence of secondary waves for these other genotypes, which followed an initial, prominent wave and its subsequent decline.

The first challenge in PCV2 history reconstruction is represented by the difficulties of ensuring a reliable and objective genotype classification, which could affect population parameter estimation. For example, strains of the newly proposed PCV2i genotype (Wang et al., 2020) had previously been classified as PCV2a, and thus the previous classification was maintained (Franzo et al., 2016a). However, since the present study was not aimed at classification definition, and to ensure that mere classification disagreements did not affect the overall picture, the analysis was repeated considering PCV2i as an independent genotype (data not shown). Although minimal variations in tMRCA and population patterns were observed, the overall pattern of PCV2a fluctuations was confirmed, demonstrating the robustness of the analysis. To further strengthen the results’ reliability, only major, robustly identified, clusters were considered, excluding small intermediate clusters, potentially ascribable to epidemiological dead ends.

The increase in sequence data allowed for a more comprehensive exploration of PCV2 genetic variability with enhanced spatial and temporal resolution, which allows circumventing the “artefactual” effect caused by limited numbers of sequences. The present work includes thousands of sequences for each genotype, as opposed to the few tens or hundreds used in earlier studies like Franzo et al. (2016a). This reflects a more intense diagnostic and sequencing effort, as well as increased representativeness of the available sequences on a global scale.

Although a strong bias toward high-income countries persists, the inclusion of data from regions and countries where pig farming practices are characterized by different, often less advanced management and less effective control measures has expanded the host population considered in this study. These ecological niches may have contributed to the maintenance and expansion of PCV2 clades that exhibited a fitness disadvantage in more industrialized countries and were thus largely overlooked in previous molecular biology studies (Molini et al., 2021; Faustini et al., 2023). This evidence is particularly suggestive considering the hypothesis that certain PCV2 genotypes may have benefitted from the intensification of the pig farming sector and the introduction of PCV2 vaccines, while others were able to persist in more restricted ecological niches. The essentially stable population estimated for minor genotypes, which exhibited a restricted geographical distribution and occasionally circulated within isolated host populations, may support this observation.

However, the availability of sequences alone can hardly account for the complexity of the observed patterns. Epidemiological factors, like viral dispersal and variable population immunity over time and geographical areas, also play a significant role. Evidence supporting this comes from the comparison of results obtained through the analysis of two random datasets generated using alternative approaches. In fact, selecting up to four sequences per year-country pair has been shown to yield consistent results across repeated trials in previous studies. This method is considered effective and epidemiologically sound, especially when the number of available sequences is influenced by biases in strain collection or sequencing activities over time and geographical area, rather than by actual differences in strain or genotype circulation across various countries or time periods. In scenarios where true variability exists, enforcing an equal sample size for each country-year pair could disproportionately highlight countries with lower viral circulation (that could have been considered responsible for the detection of multiple waves). To address this potential issue, a random subset with the same composition as the original one was generated—excluding several of the rare country-year pairs—analyzed using the same methodology, and compared to the original results (Hall et al., 2016; Layan et al., 2023). Findings were largely consistent, suggesting that the sequencing bias or subsampling strategy had minimal impact. This consistency underscores the approach robustness even in the face of inevitable study limitations, and confirms that the estimated persistence of PCV2a and PCV2b at non-negligible levels reflects actual epidemiological and evolutionary patterns. Accordingly, the reconstruction of lineages over time supports the progressive expansion and more balanced distribution of all major genotypes at the global level, with increasingly comparable frequencies across the studied regions. While Asia, Europe, and North America continue to show the most significant involvement, South America and Africa are also affected, albeit to a lesser extent. Particularly in the case of Africa, the limited availability of samples likely influenced the results. The inclusion of nearly all available African sequences in the Country-year dataset revealed a more substantial involvement of this region than previously recognized. Given the rising importance of Africa in pig production and its growing economic and commercial relations with other regions, especially Asia, there is an urgent need to gain a better understanding of the epidemiological patterns of PCV2 in Africa (Franzo et al., 2023; Dundon et al., 2024).

Similarly, the evaluation of migration events through phylogeographic analysis confirms the remarkable global dispersal of PCV2, characterized by multiple introduction events into new areas, followed by local persistence. Given the likely competition among distinct genotypes and clades—driven by limited host availability and differential cross-protection between homologous and heterologous strains—the periodic introduction of new strains, independently evolved in different environments, may have provided a competitive advantage to these newly colonizing strains.

It is important to emphasize that, based on current knowledge, there is no consistent evidence of significant differential clinical cross-protection among genotypes, and most cases of so-called vaccine failure are likely attributable to improper vaccination management (Franzo and Segalés, 2020; Sibila et al., 2021; Segalés and Sibila, 2022). However, there is substantial evidence indicating lower neutralization capabilities of heterologous immunity, as well as higher levels of viremia and viral transmission documented both experimentally and epidemiologically (Huang et al., 2011; Liu et al., 2013; Opriessnig et al., 2013a; Dvorak et al., 2016). Therefore, while not clinically significant, even a marginal differential in cross-protection, when acting on large viral populations, could have impacted the epidemiological and evolutionary landscape.

Whereas previous understanding suggested the periodic emergence of entirely new genotypes, the scenario identified here points more toward periodic fluctuations of currently circulating genotypes. This might be the result of an ongoing evolutionary “arms race” involving different PCV2 clades, with the global pig population serving as the battlefield. The heterogeneous and dynamically changing immunity of this population defines the selective pressures, thereby shaping the viral fitness landscape. According to the observed scenario, we speculate that genotypes with greater biological fitness tend to expand and dominate. However, the rise in prevalence of these dominant genotypes generates immunity in the population, which may drive selective pressures leading to the emergence of new genotypes since the differential cross-protection at the epidemiological scale may confer them a fitness advantage. This selective pressure likely contributed to the replacement of PCV2a by PCV2b, and later, of PCV2b by PCV2d. Vaccination efforts may have further accelerated this process, as immunity against PCV2a strains may have been less effective against PCV2d, facilitating its spread. However, the reduced circulation of PCV2a and PCV2b, combined with the emergence of strains featured by an altered amino acid profile, might have decreased the effectiveness of pre-existing immunity, allowing new wave emergence. Geographical variability and the periodic introduction of new strains with different evolutionary histories may contribute to local differences in genotype fitness. This evolutionary process may represent an example of balancing selection, specifically negative frequency-dependent selection, where a genotype’s fitness diminishes as it becomes more common, and vice versa. While experimental approaches could be considered to test these hypotheses, replicating the complex epidemiological dynamics in a controlled setting presents significant challenges.

The role of environmental conditions and contingencies in affecting PCV2 genotypes’ success, emerges also by the tMRCA estimation of PCV2b and PCV2d. While PCV2d was initially considered a PCV2b-mutant (Opriessnig et al., 2013b) the divergence between these two genotypes is likely more ancient than previously thought and co-circulated for several years or decades before emerging as major genotypes, as revealed by present and previous phylodynamic and molecular epidemiology studies (Franzo et al., 2016a).

The pivotal role of host differential immunity was consistently confirmed by all methods used to investigate the characteristics of selective pressures. Most of the detected sites were subjected to episodic rather than diversifying selective pressures, indicating that selection occurs through selective bursts. This scenario aligns well with the hypothesis of the periodic emergence of new genotypic and phenotypic groups that adapt to a changing immune environment, followed by stabilization once a higher fitness phenotype is achieved.

The stronger diversifying selection and most sites under statistically significant positive selection were predominantly located in solvent-accessible regions or on the surface of the capsid protein. Notably, many of these sites correspond to capsid positions previously identified as linear or conformational epitopes in experimental studies, including some recognized as affecting genotype-specific recognition by monoclonal antibodies (Mahé et al., 2000; Lekcharoensuk et al., 2004; Shang et al., 2009; Trible et al., 2011; Saha et al., 2012a; Saha et al., 2012b). This result suggests that the evolutionary pressure exerted by host immunity is particularly focused on these exposed regions, driving the adaptation and evolution of the virus in response to immune challenges.

The detection of different sites under significantly different selective pressures among genotypes confirms that PCV2 cannot be considered a uniform entity from an immunological perspective. Distinct selective forces, varying in both location and intensity, act on different genotypes. While it is challenging to determine whether these differences are attributable to structural variations in the capsid or to peculiarities in local host population immunity, the multifactorial nature of PCVDs and its deep interaction with the host suggests that a combination of viral and host factors, but also environmental ones (co-infections, nutrition, management, animal movements, biosecurity, control strategies, and others), are likely to be involved.

Notably, the analysis of amino acid frequency in submitted sequences over time revealed a tendency for different genotypes not to display the same predominant amino acid in epitopic sites. For instance, an increase in the frequency of a particular amino acid or the emergence of a new genotype characterized by a specific amino acid at a given capsid position is sometimes followed by a parallel substitution or decrease in the frequency of that same amino acid in other genotypes. This pattern may be explained by the rise of epitope-specific immunity, which could temporarily decrease the prevalence of a genotype, followed by a resurgence when a new partially immune-escaping profile emerges. This dynamic interplay between viral evolution and host immune pressure underscores the complexity of PCV2 adaptation and persistence within different host populations.

While a direct comparison of the amino acid positions identified in this study with those determined experimentally might be of limited utility—due to the significant differences in the binding of monoclonal antibodies to specific strains *in vitro* compared to the effects of polyclonal sera acting on a wide array of antigens in a broad population at the epidemiological level—some important considerations can still be made. First, more pronounced fluctuations in the amino acid profiles were observed in PCV2a, particularly from 2005 onwards, which might be linked to the introduction of vaccines. However, similar fluctuations continued to occur thereafter, suggesting the influence of immunity induced by other genotypes as well. Selective pressure analysis and visual inspection of amino acid patterns over time identified a tendency to replace amino acids characteristic of other genotypes. This was notably observed at positions 63, 76, 126, 131, 134, and 190. Similar phenomena, albeit to a lesser extent, were also observed in PCV2b and PCV2d, mainly after 2010. Position 59 is particularly noteworthy, as a decrease in the presence of arginine (R) was observed in PCV2a Cap protein following the emergence of PCV2b, which Cap protein is characterized by this amino acid. Concurrently, PCV2d experienced a decrease in the occurrence of alanine (A), which was the dominant amino acid in PCV2a Cap protein. Both PCV2b and PCV2d showed an increase in the frequency of lysine (K) at position 59 after 2010. While this convergence might seem counterintuitive, lower protection conferred by the R59K mutation has been reported in mutagenesis studies and previously suggested in molecular epidemiology studies as a PCV2a vaccine escaping mechanism (Franzo et al., 2016b; Huang et al., 2020). An evolutionary advantage might, therefore, explain the convergence toward specific amino acids in certain positions. A comparable pattern was observed at position 190 T.

## 5 Conclusion

The results of the present study confirm previous evidence of the alternation of major genotypes on the epidemiological stage and highlight the role of inter-genotype competition and host immunity—including vaccine-induced immunity—in shaping the evolutionary success of specific strains and genotypes. However, the updated and more geographically and temporally representative sequence dataset suggests a negative frequency-dependent selection, allowing the continuous circulation of major genotypes at varying levels over time, rather than the complete genotype replacement initially hypothesized (the so-called “genotype shifts”). This is evidenced by the persistent detection of PCV2a and PCV2b, as well as the less frequently found genotypes, at the time PCV2d is the dominating genotype over the world.

While the determinants of this scenario are challenging to fully elucidate, the present analysis indicates that variations in genotype- or clade-specific immunity—affected by the local prevalence of viral groups—combined with the periodic introduction of strains that have independently evolved in different environments, may have led to periodic fluctuations in the population dynamics of major genotypes over time. These fluctuations are associated with ongoing evolution and variation in the capsid amino acid profile.

This evidence has profound implications for future control strategies. While PCV2d remains the most prevalent and widespread genotype, other genotypes should not be neglected. Efforts should likely focus on fostering broader and more cross-protective immunity rather than targeting a specific genotype. Such an approach would aim not only to prevent clinical outbreaks but also to constrain the evolutionary potential of PCV2.

## Data Availability

The original contributions presented in the study are included in the article/Supplementary material, further inquiries can be directed to the corresponding author.
