# Supplementary material for: An updated phylogeography and population dynamics of porcine circovirus 2 genotypes: are they reaching an equilibrium?
Source: Front Microbiol. 2024 Oct 29;15:1500498. doi: 10.3389/fmicb.2024.1500498 (PMC11554664; doi:10.3389/fmicb.2024.1500498)
Supplement: Supplementary file 1 [file Data_Sheet_1.ZIP › Supplementary figure 1.pdf]

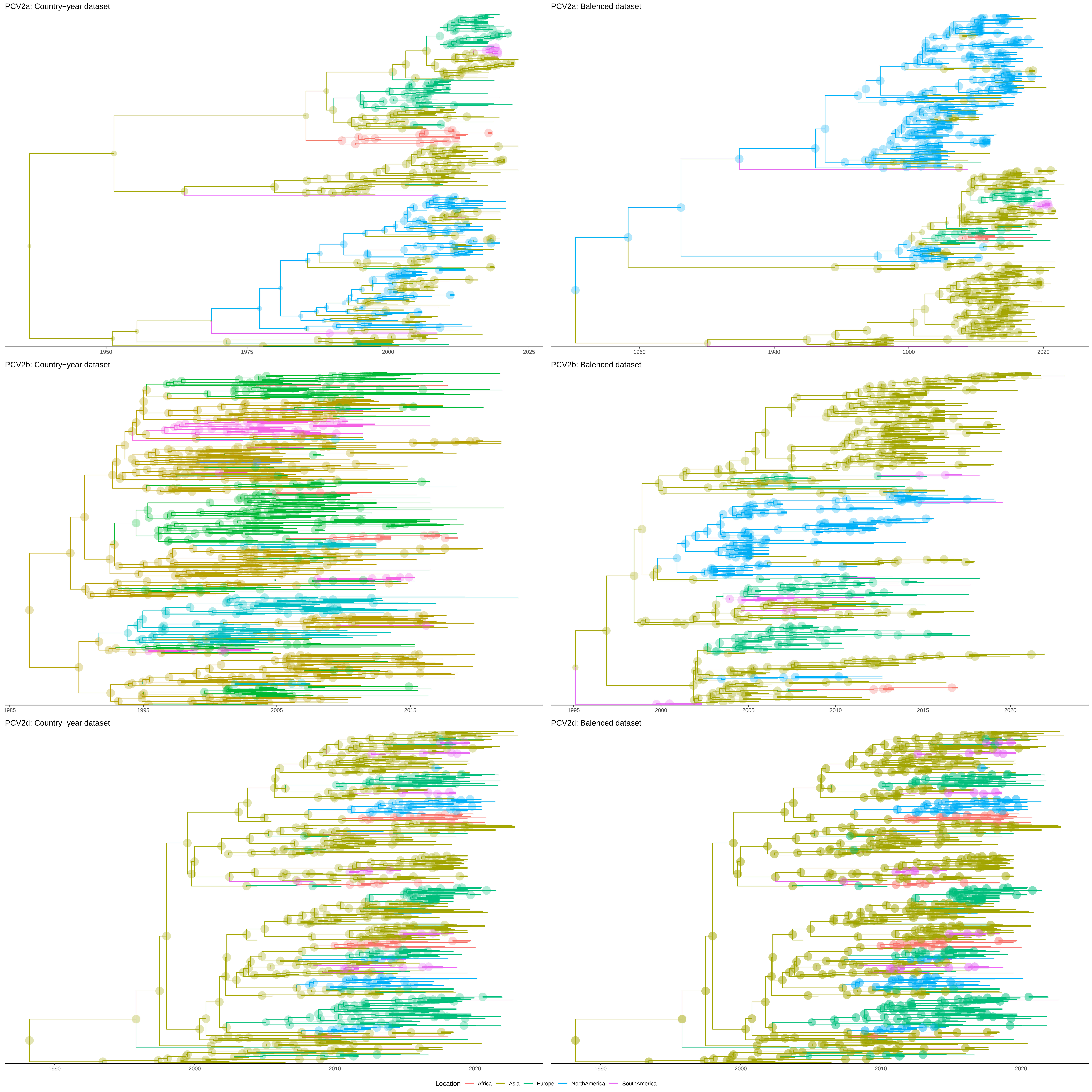
PCV2a: Country−year dataset
PCV2a: Balanced dataset
PCV2b: Country−year dataset
PCV2b: Balanced dataset
PCV2d: Country−year dataset
PCV2d: Balanced dataset
1950
1975
2000
2025
1960
1980
2000
2020
1985
1995
2005
2015
1995
2000
2005
2010
2015
2020
1990
2000
2010
2020
1990
2000
2010
2020
Location
Africa
Asia
Europe
NorthAmerica
SouthAmerica
