# Supplementary figures and images for: An updated phylogeography and population dynamics of porcine circovirus 2 genotypes: are they reaching an equilibrium?
Source: Front Microbiol. 2024 Oct 29;15:1500498. doi: 10.3389/fmicb.2024.1500498 (PMC11554664; doi:10.3389/fmicb.2024.1500498)

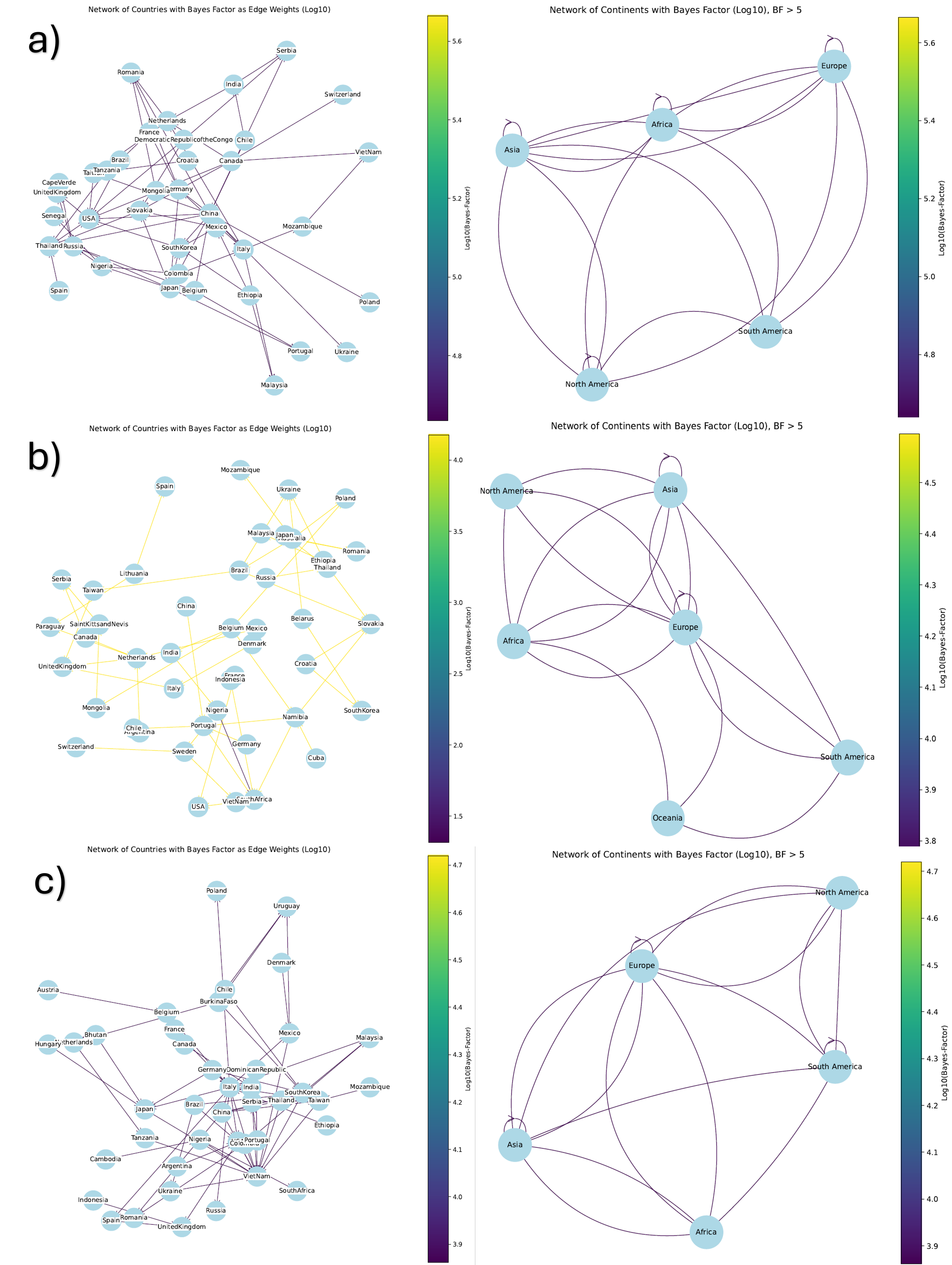

Supplement: Supplementary file 1 [file Data_Sheet_1.ZIP › Supplementary figure 2.png]

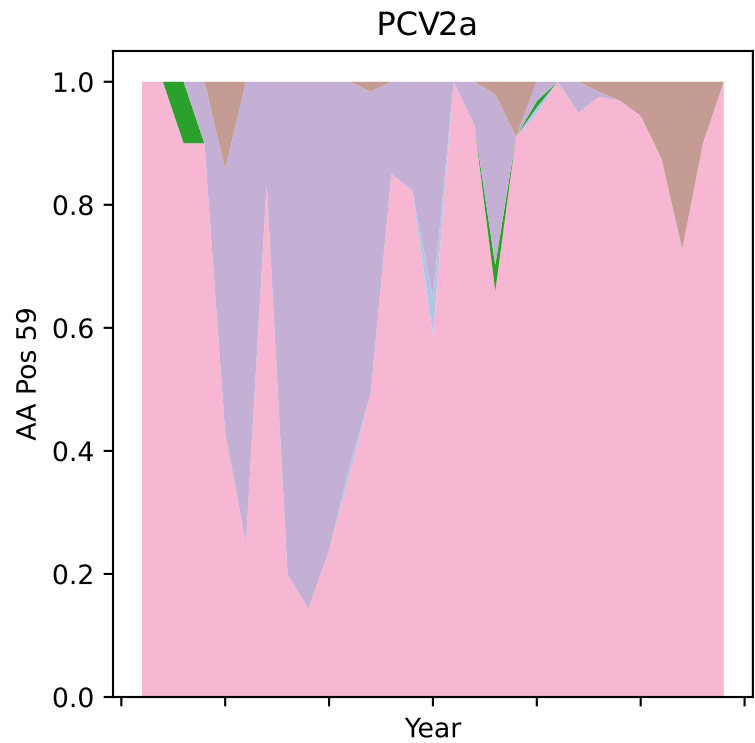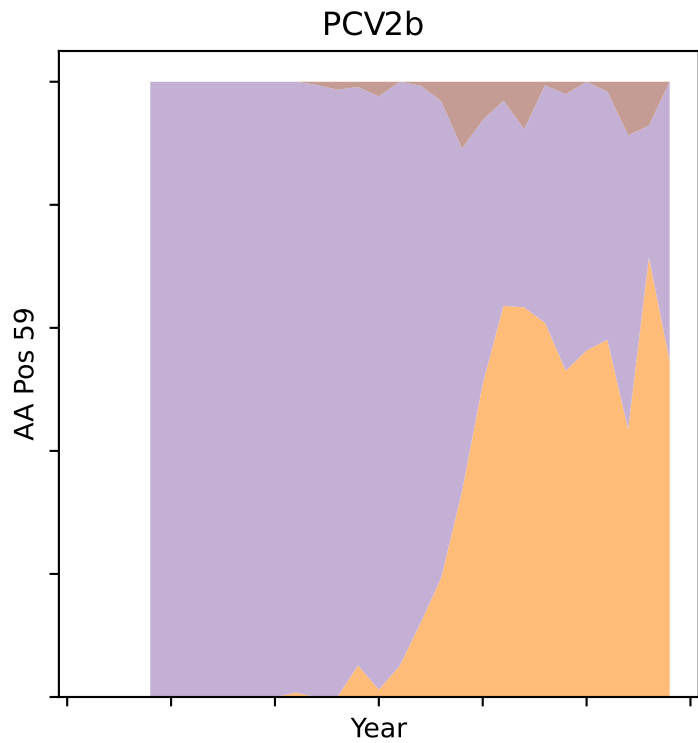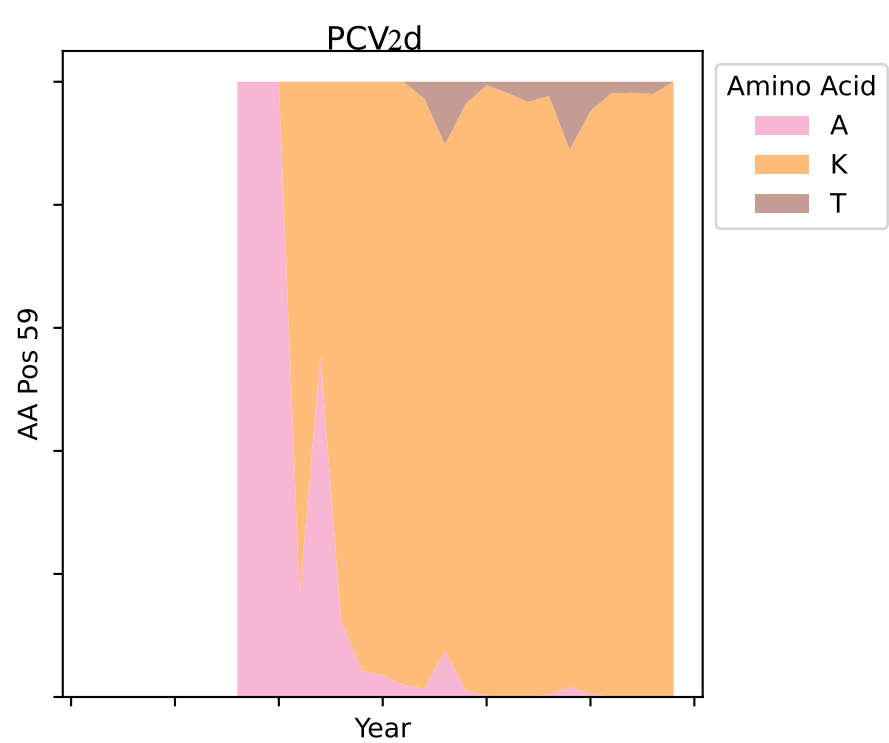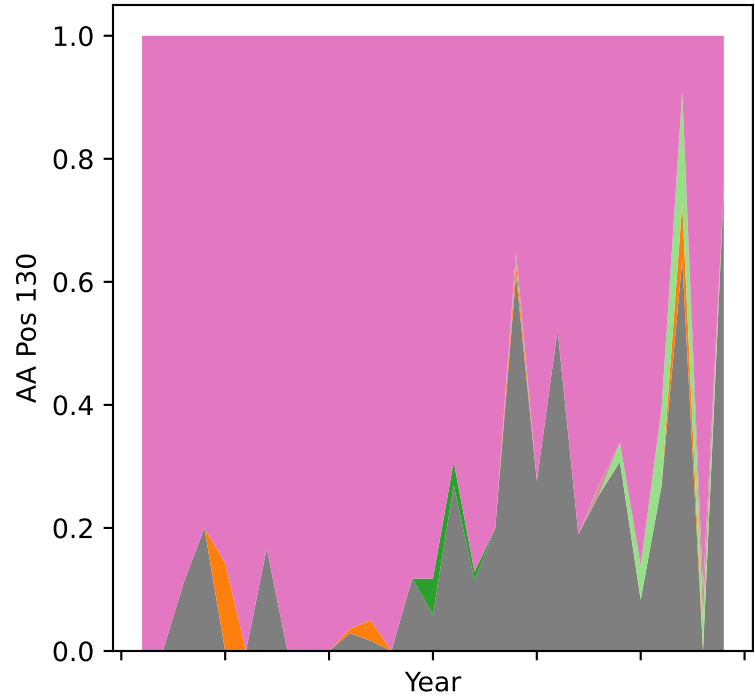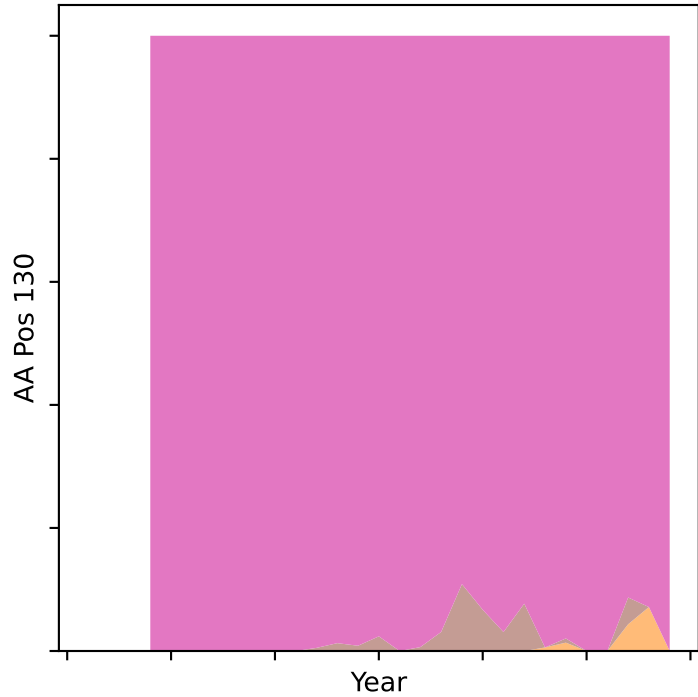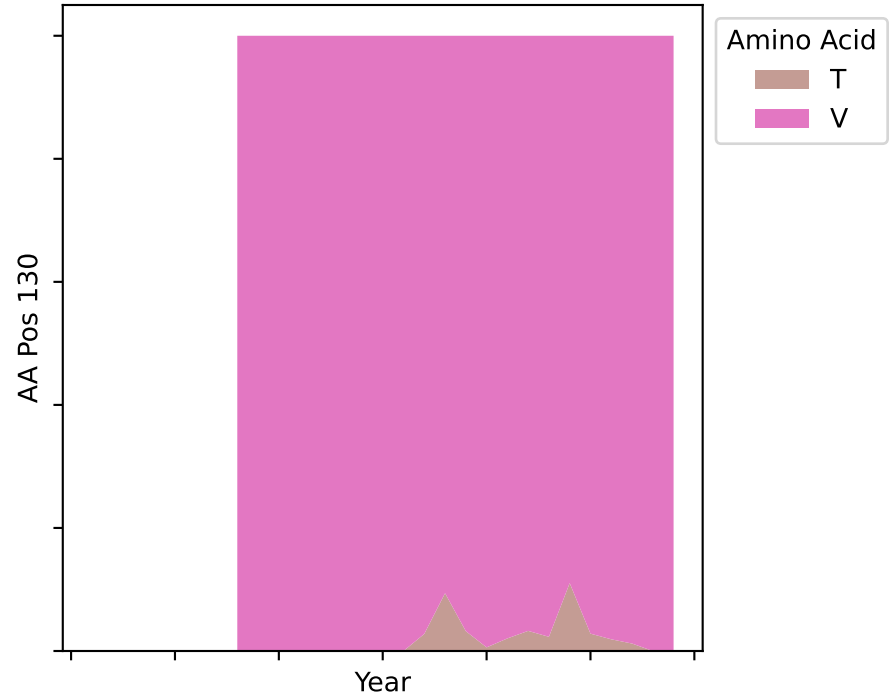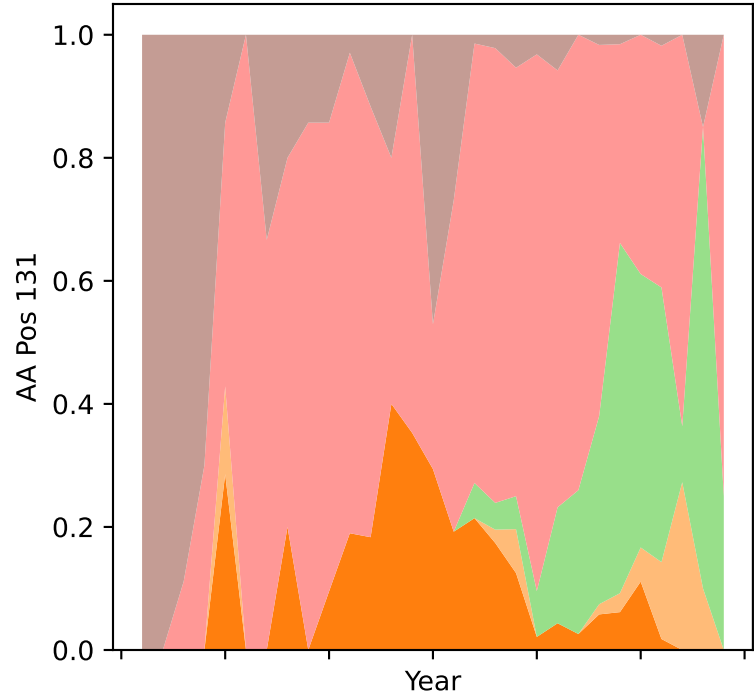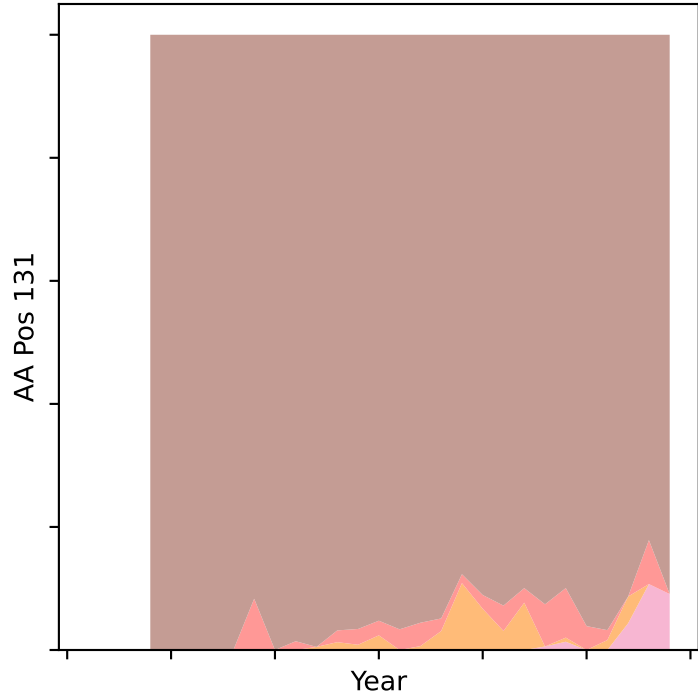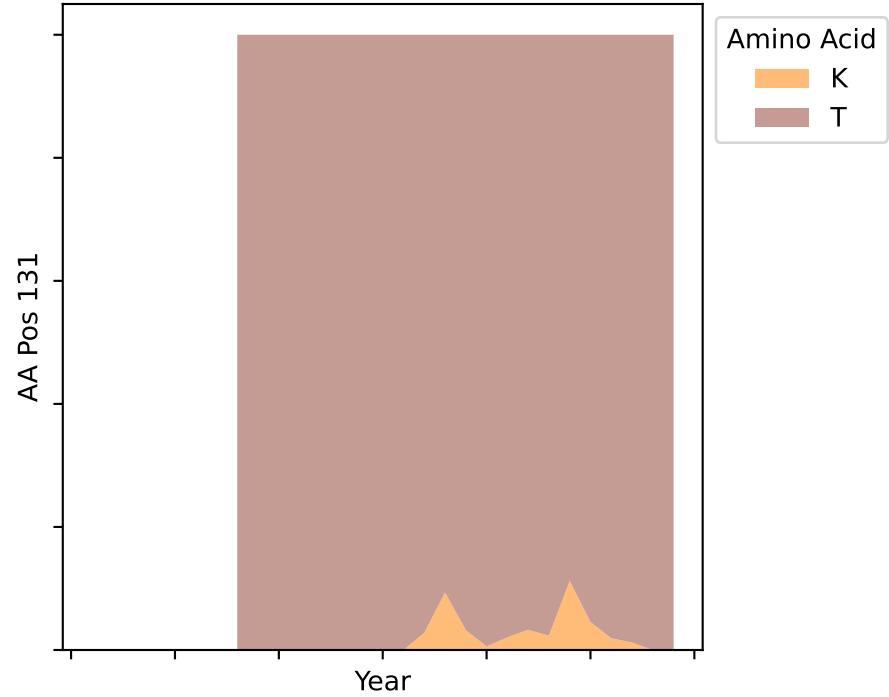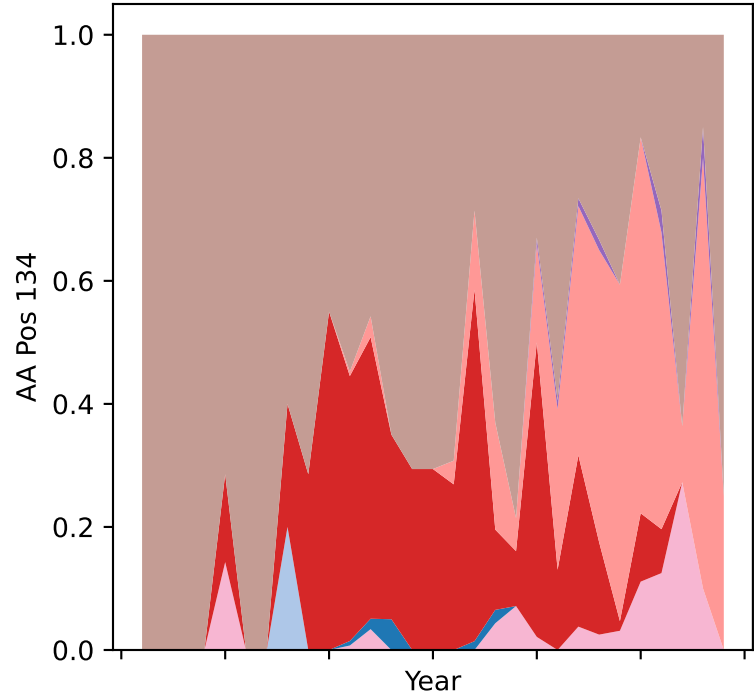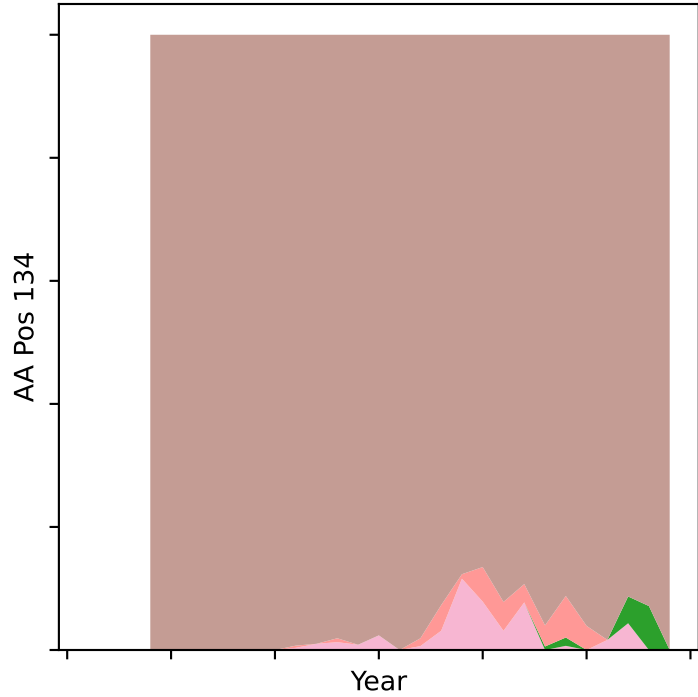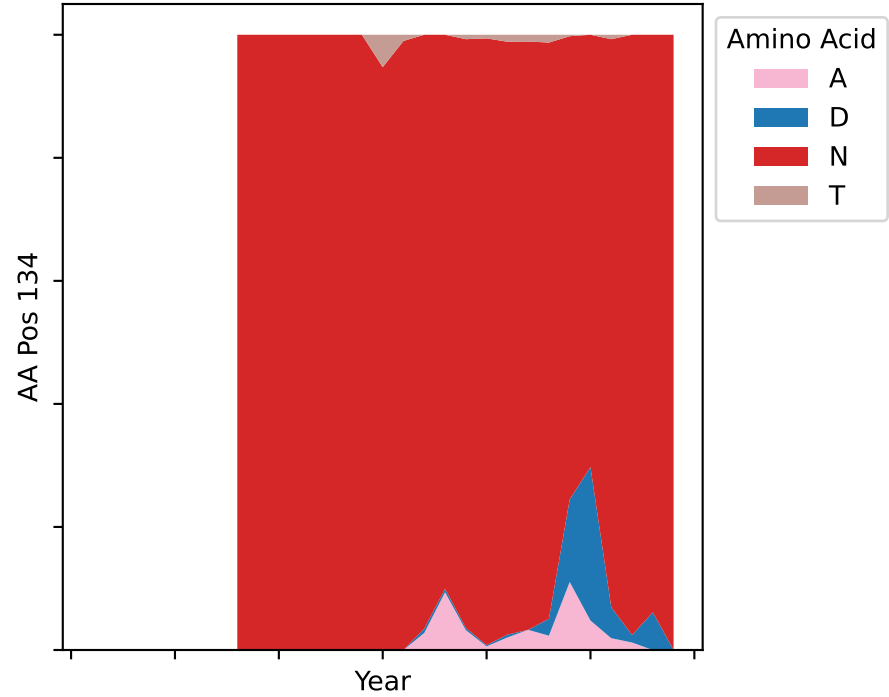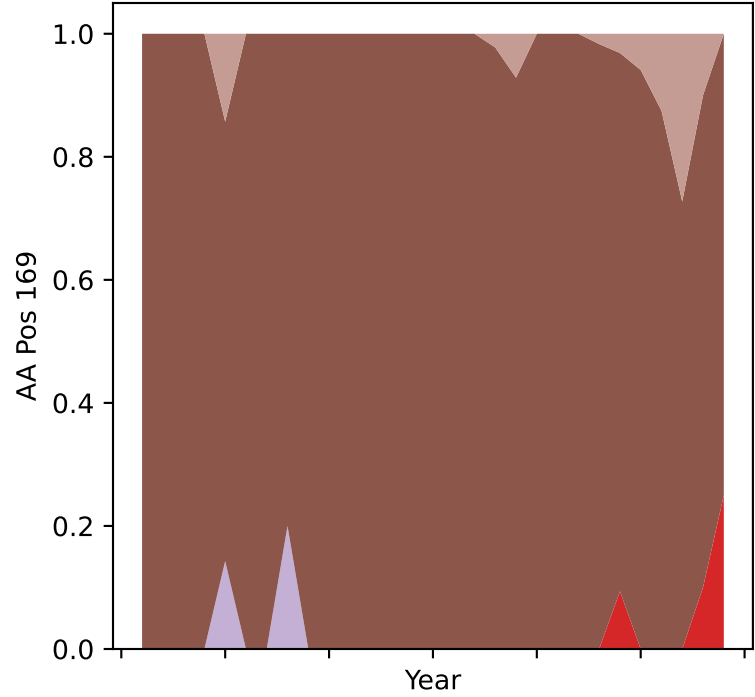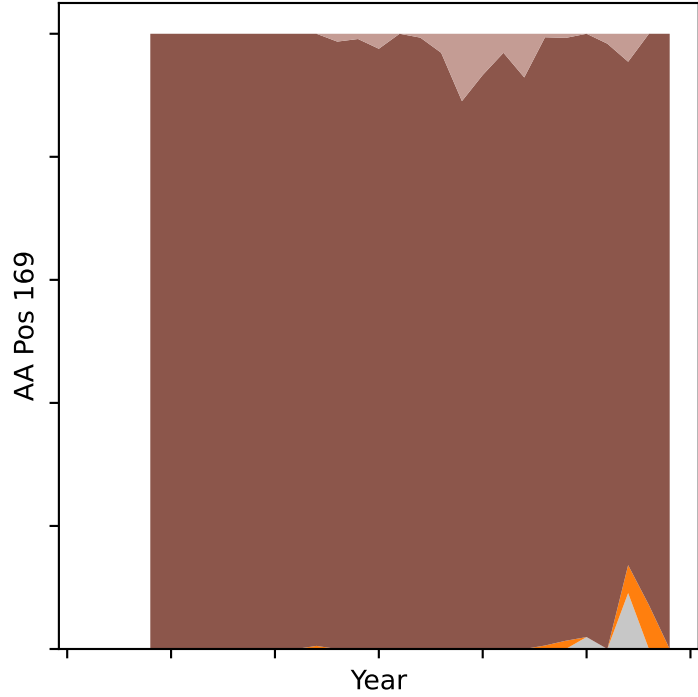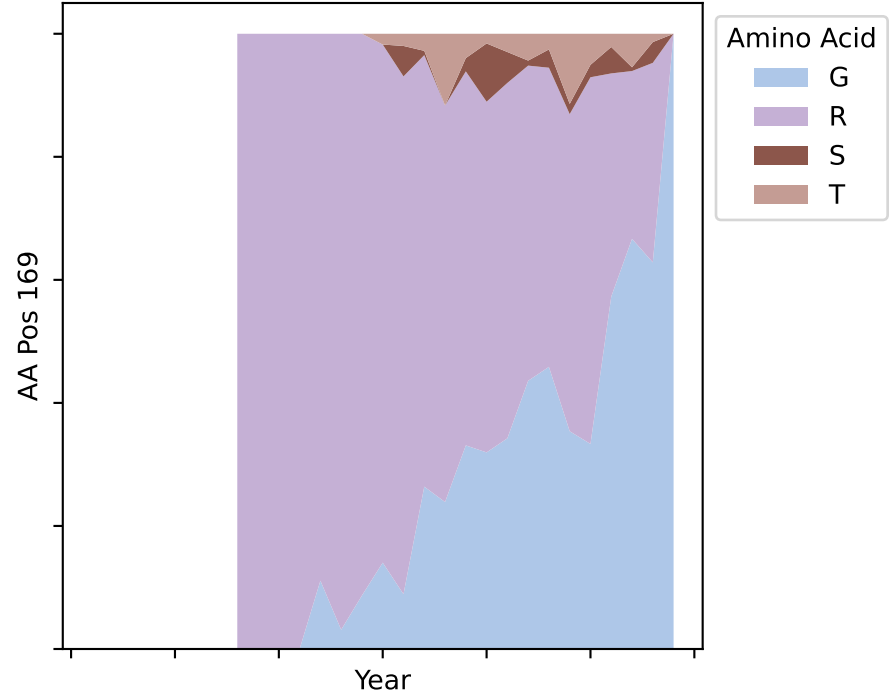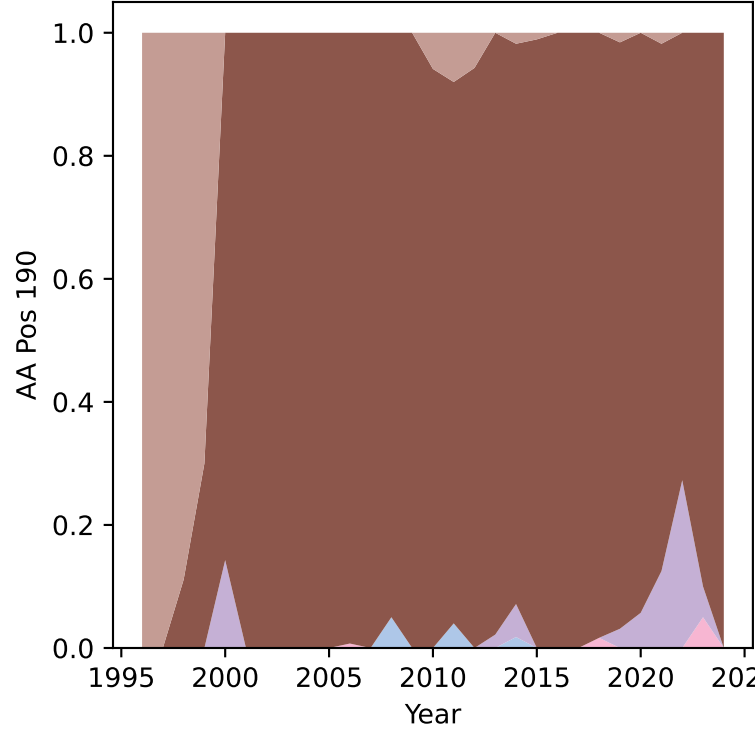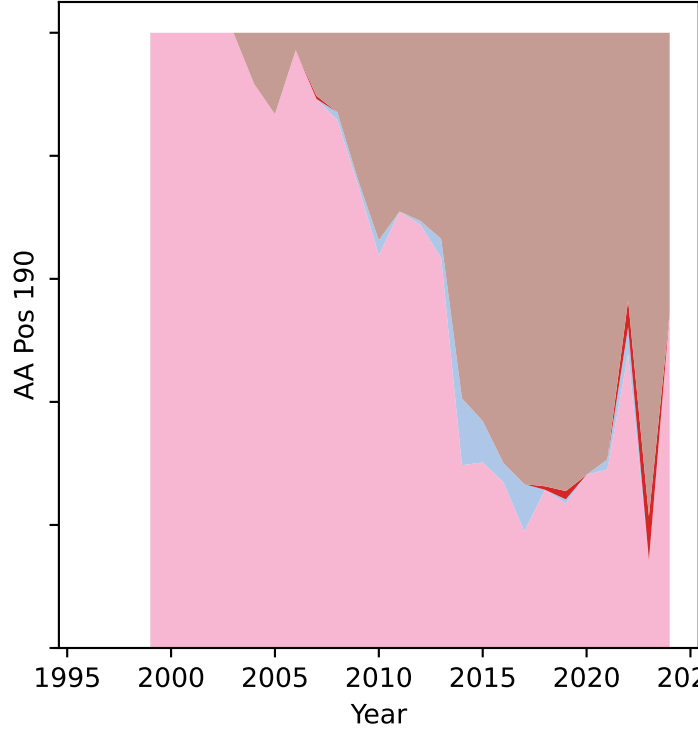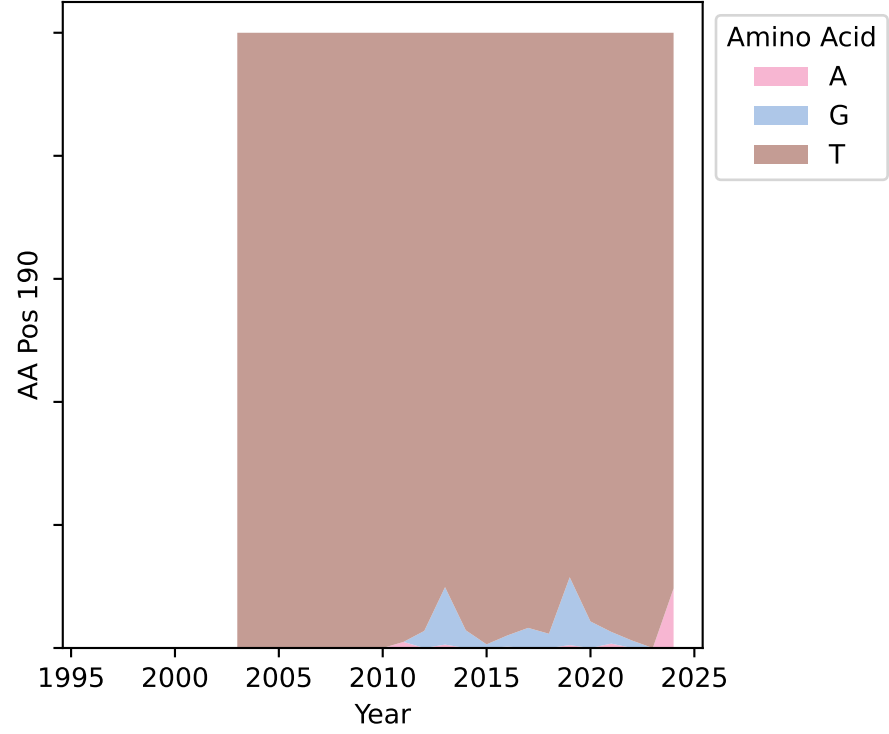

Supplement: Supplementary file 1 [file Data_Sheet_1.ZIP › Supplementary figure 3.pdf]

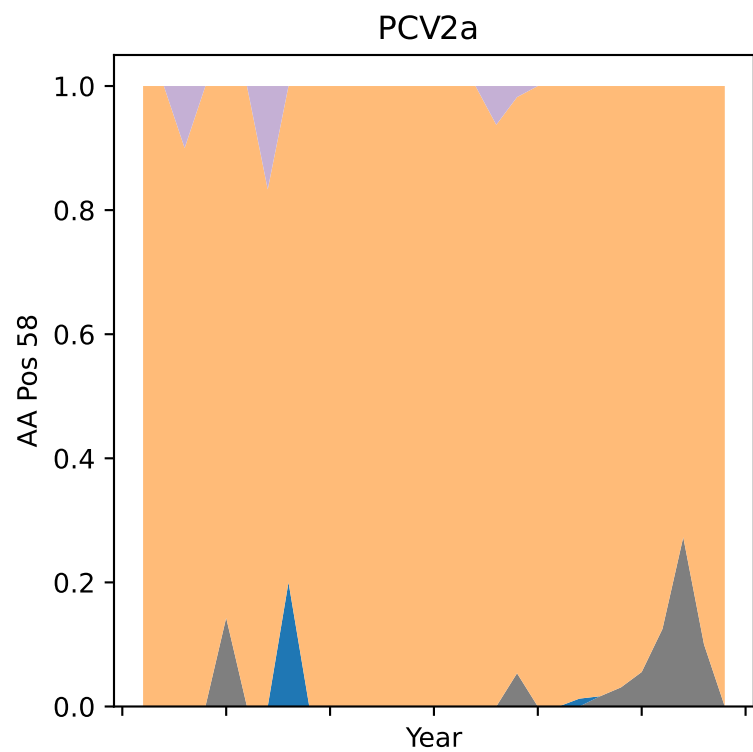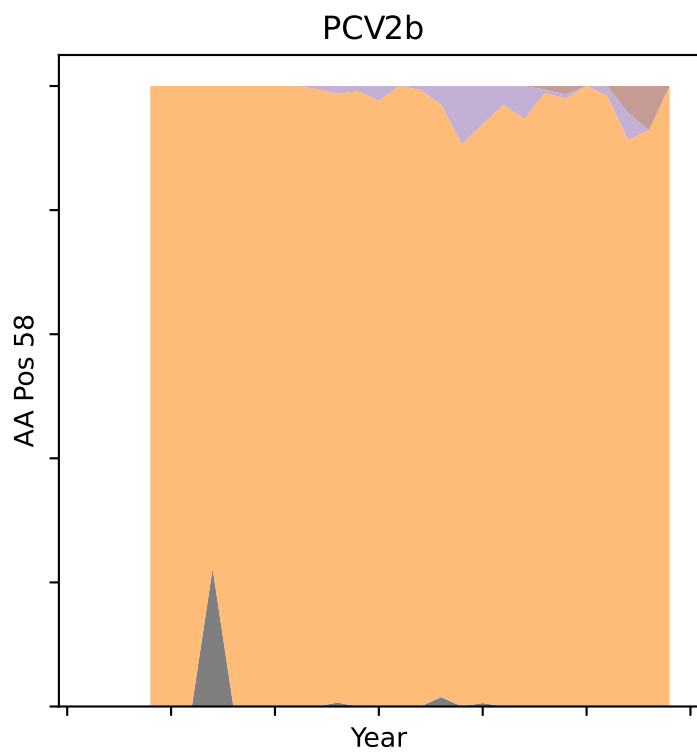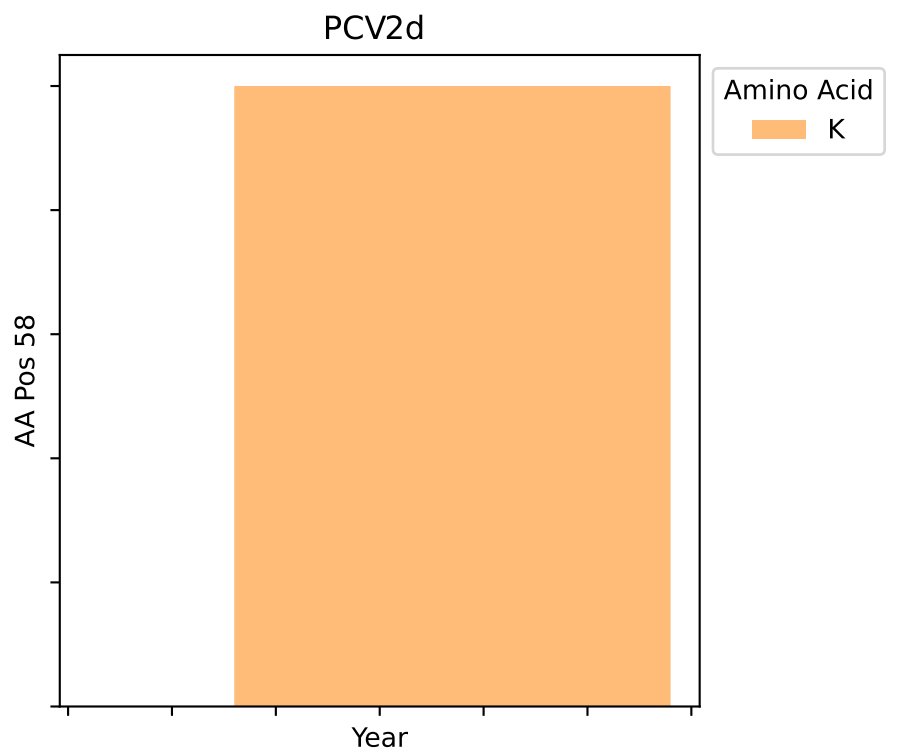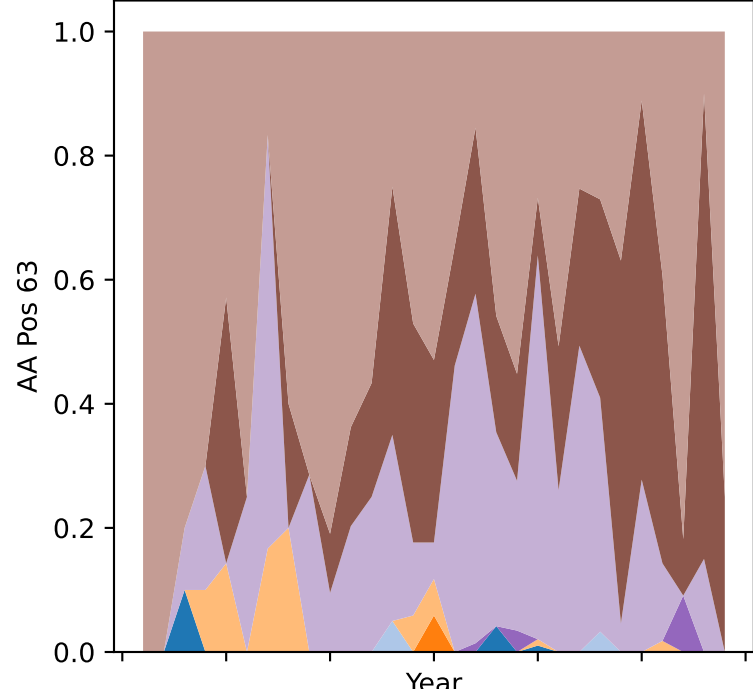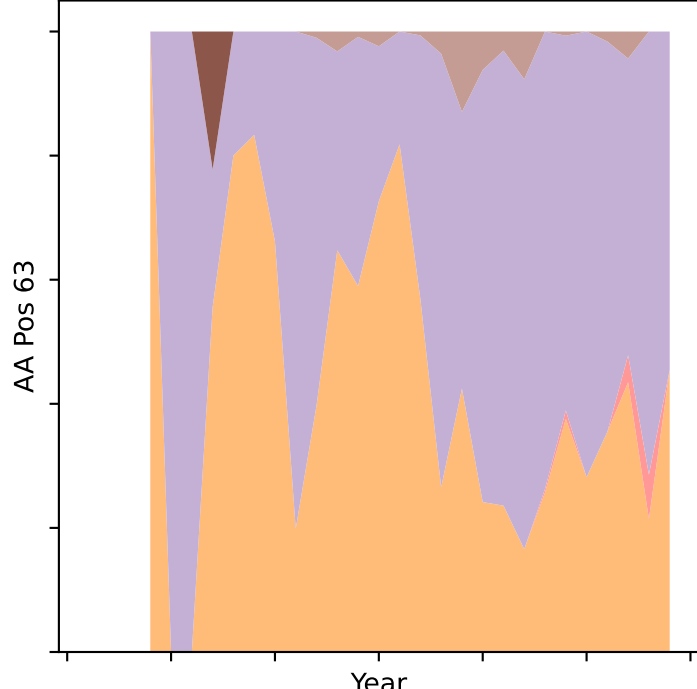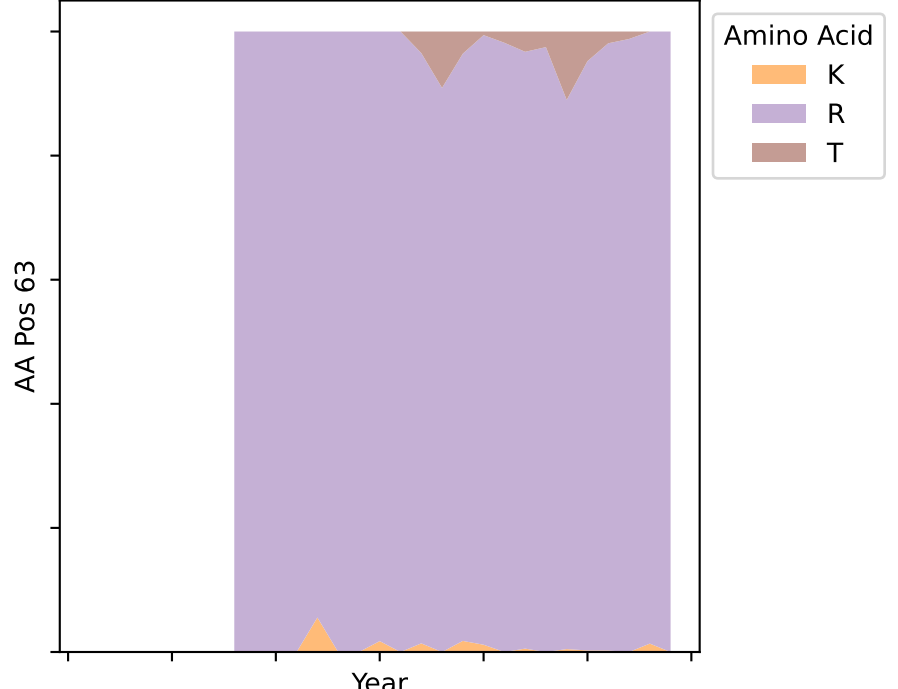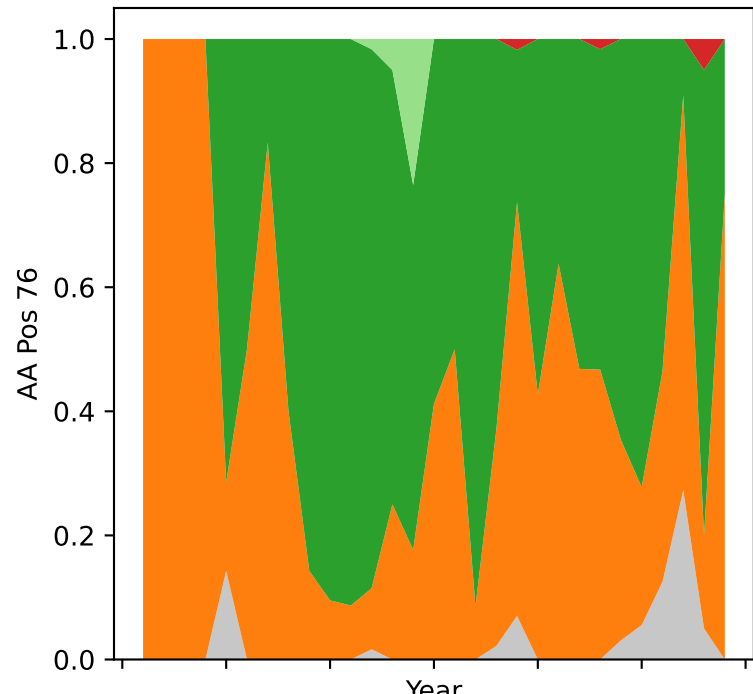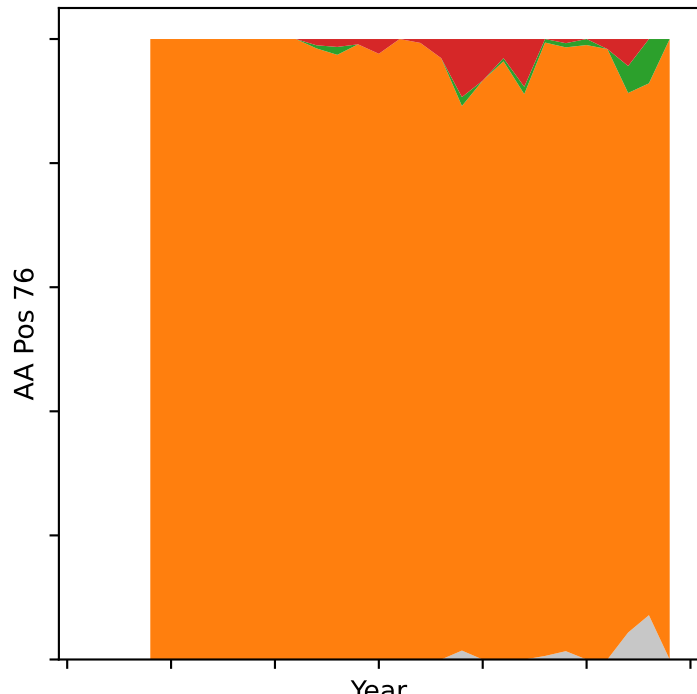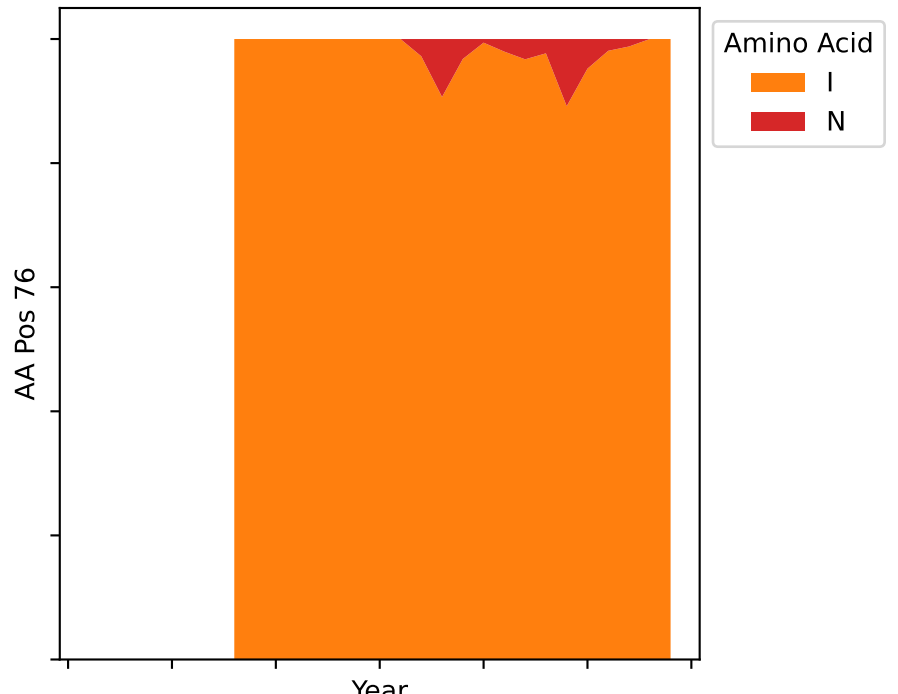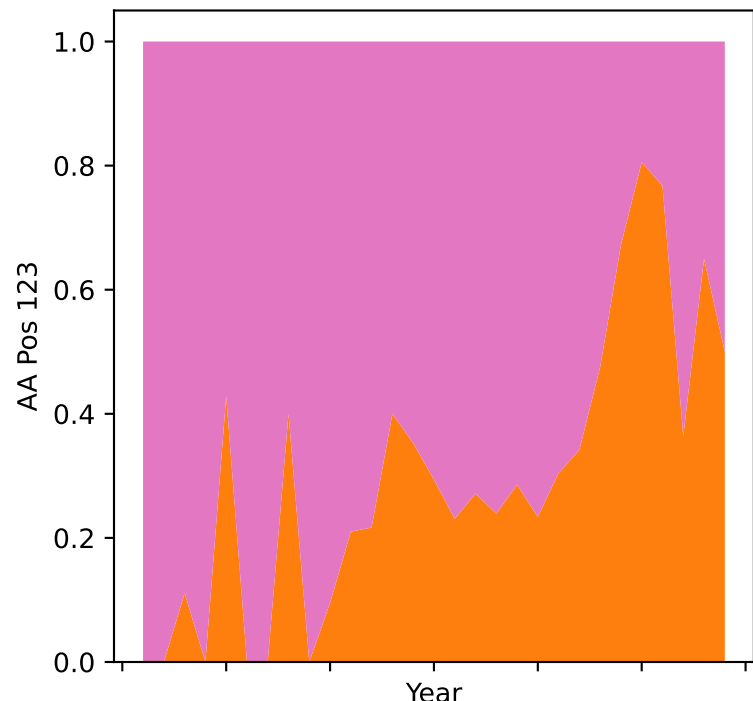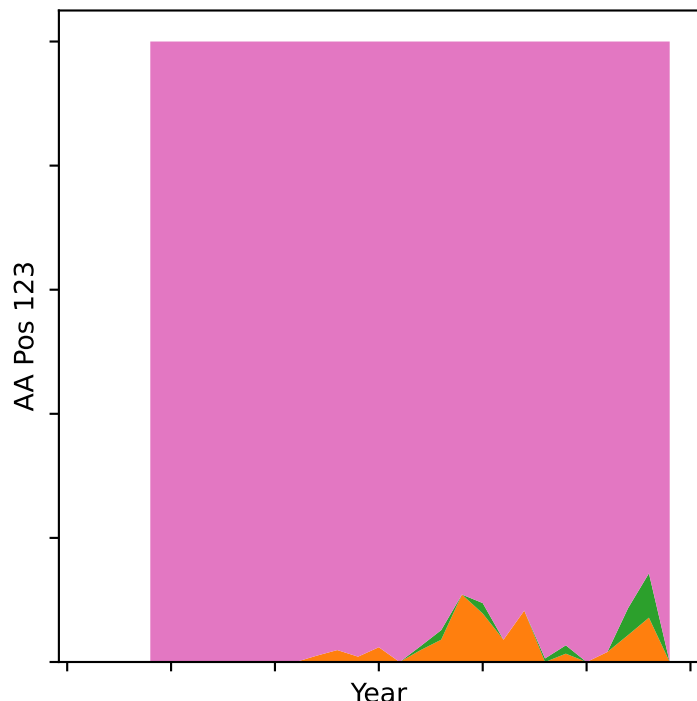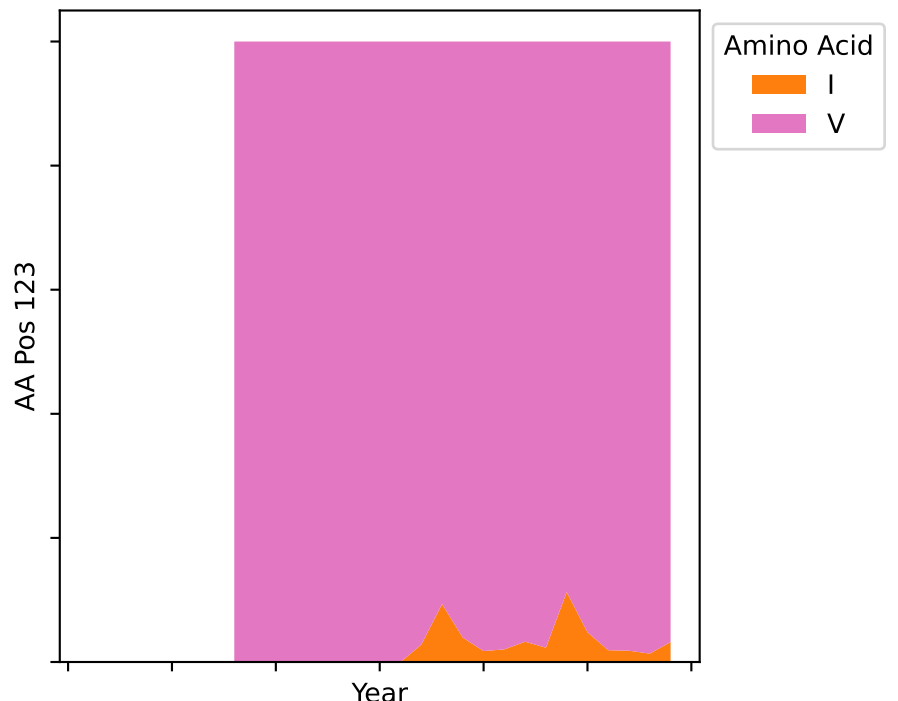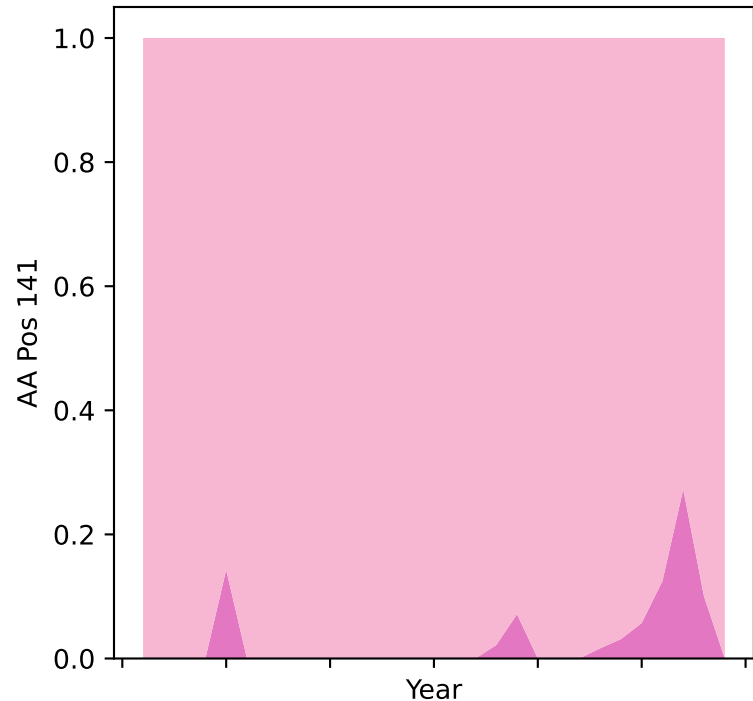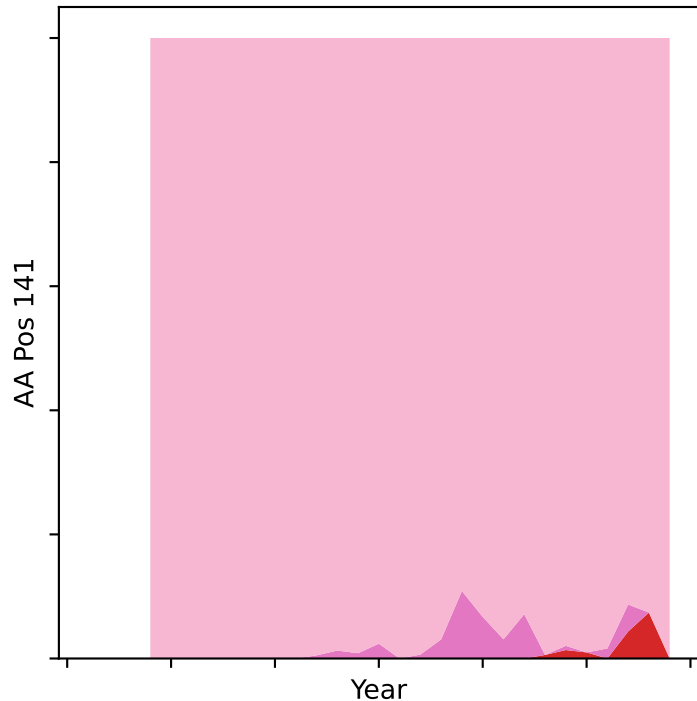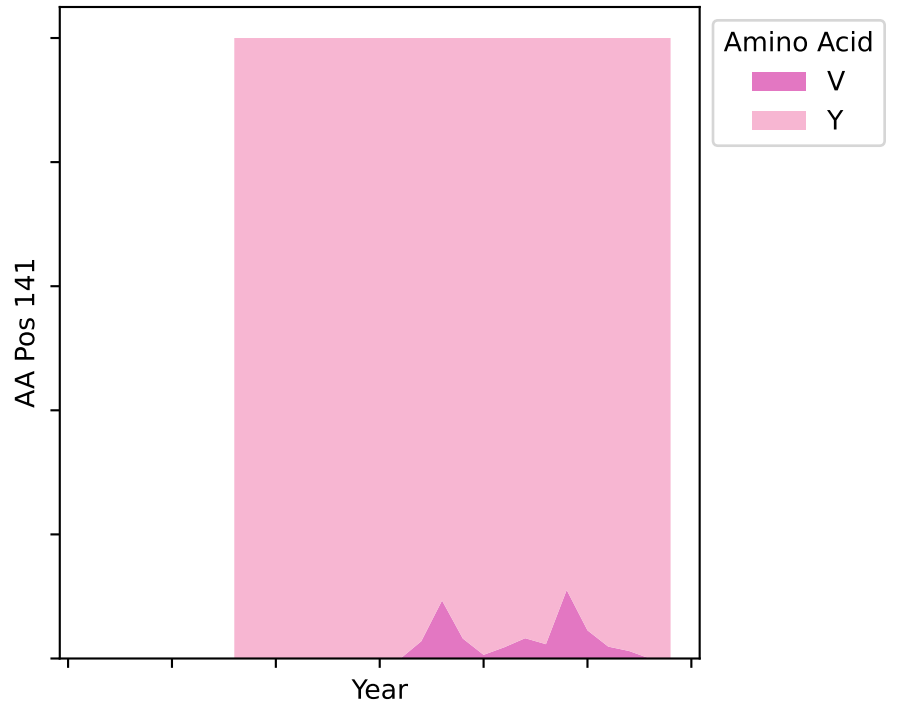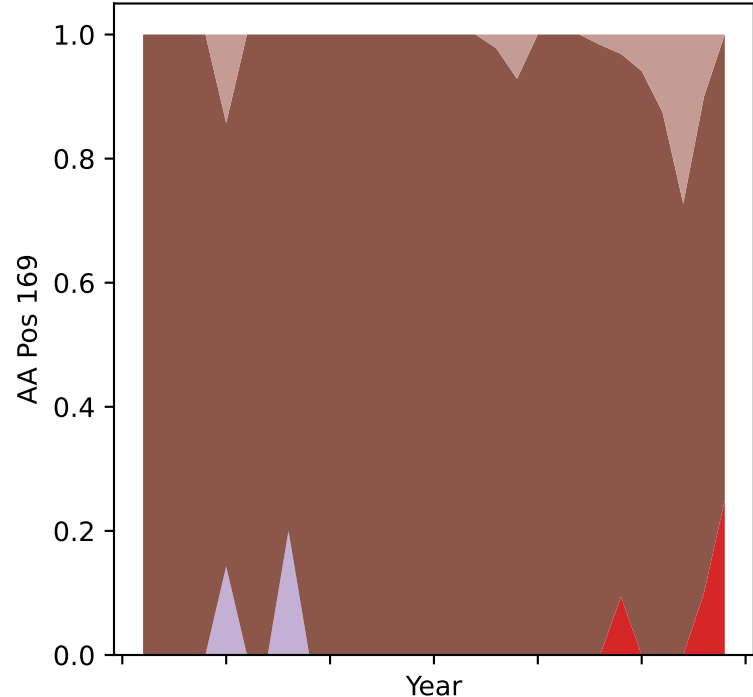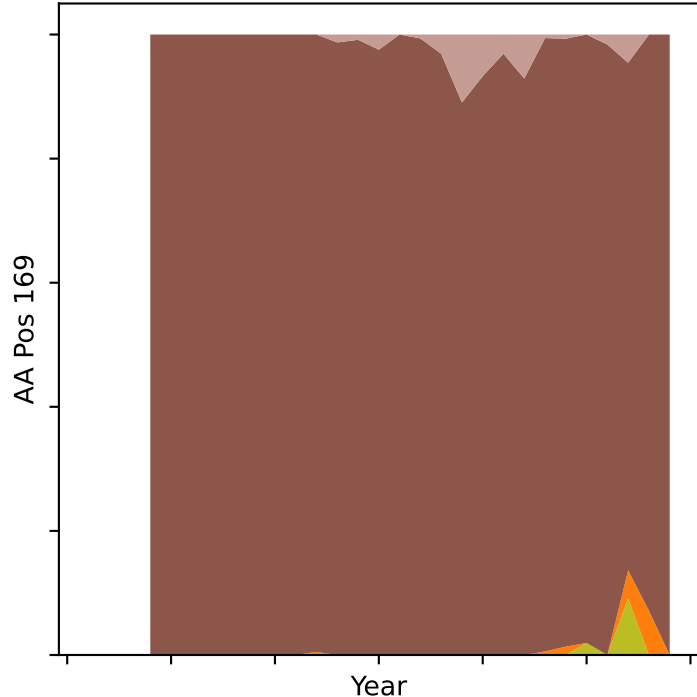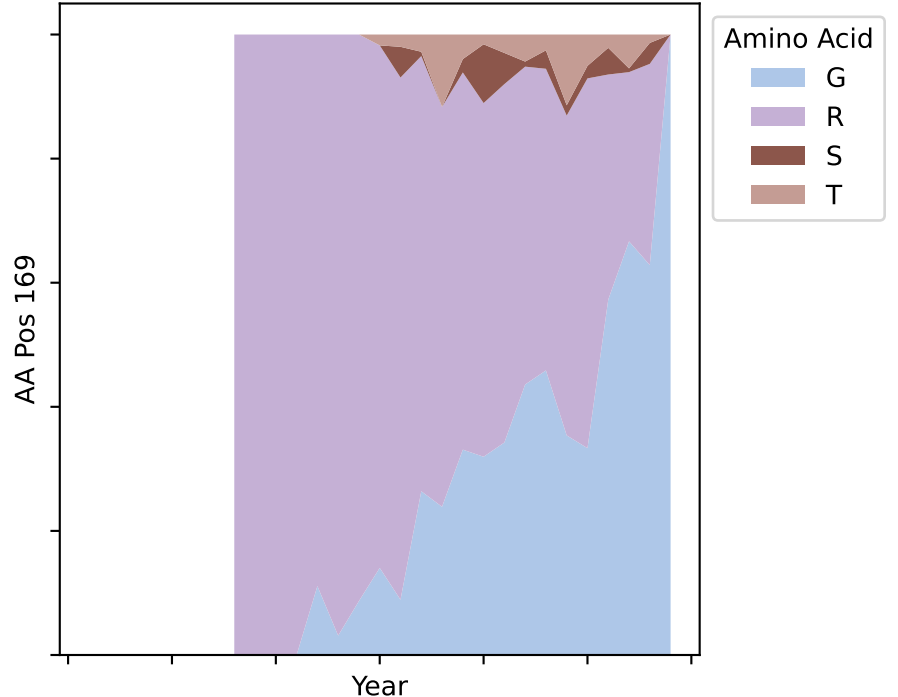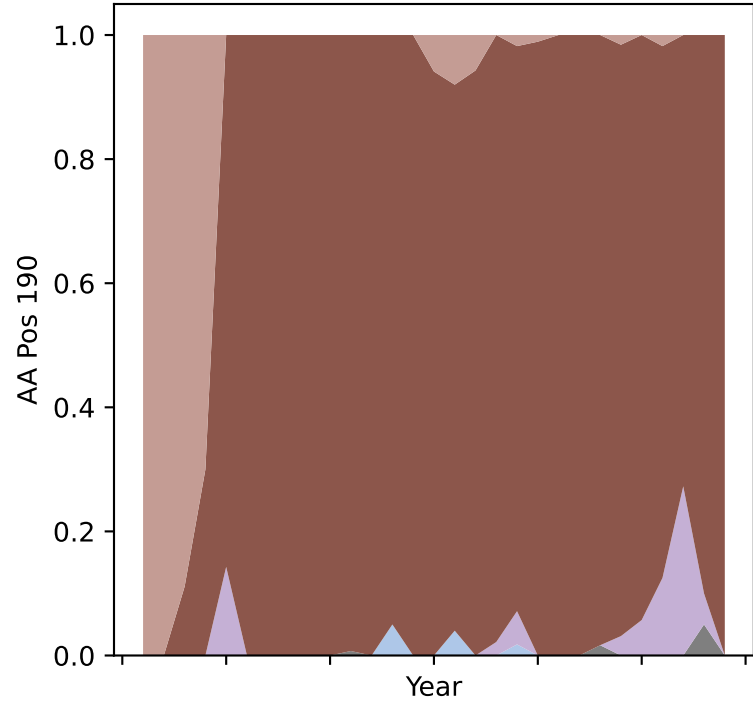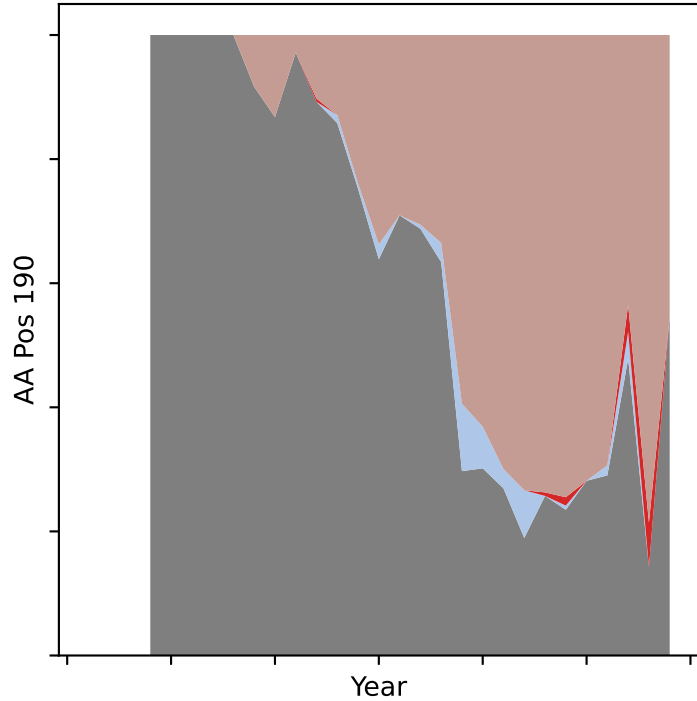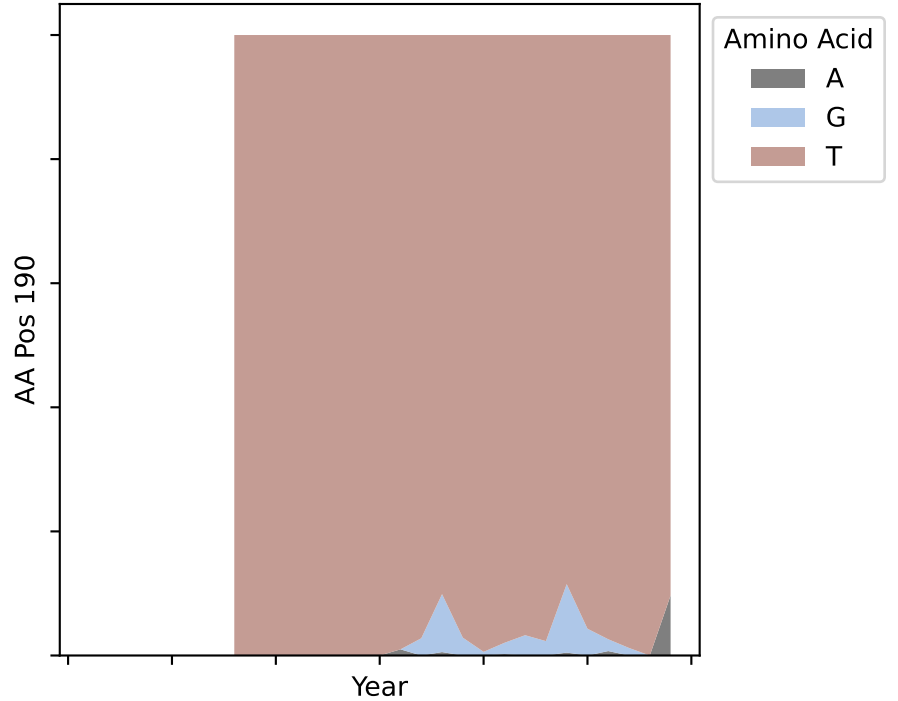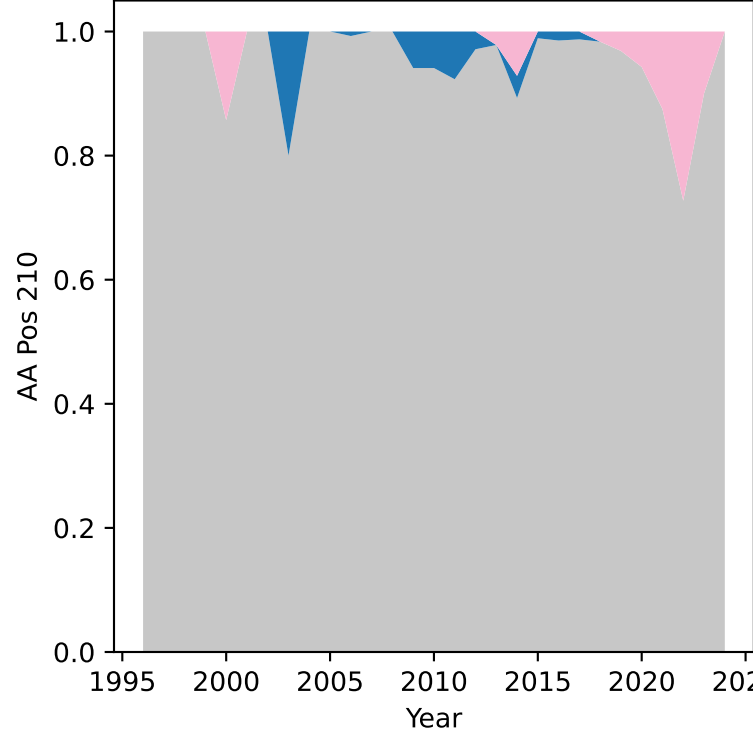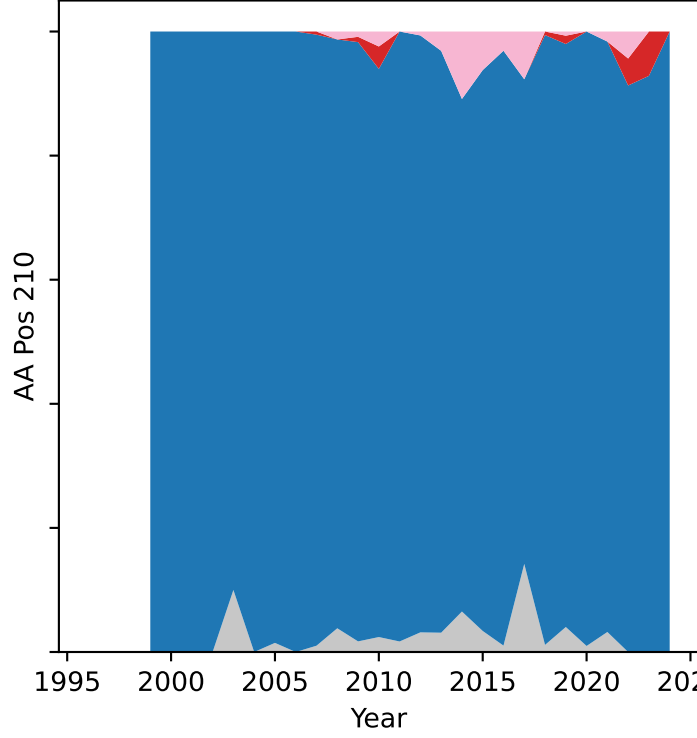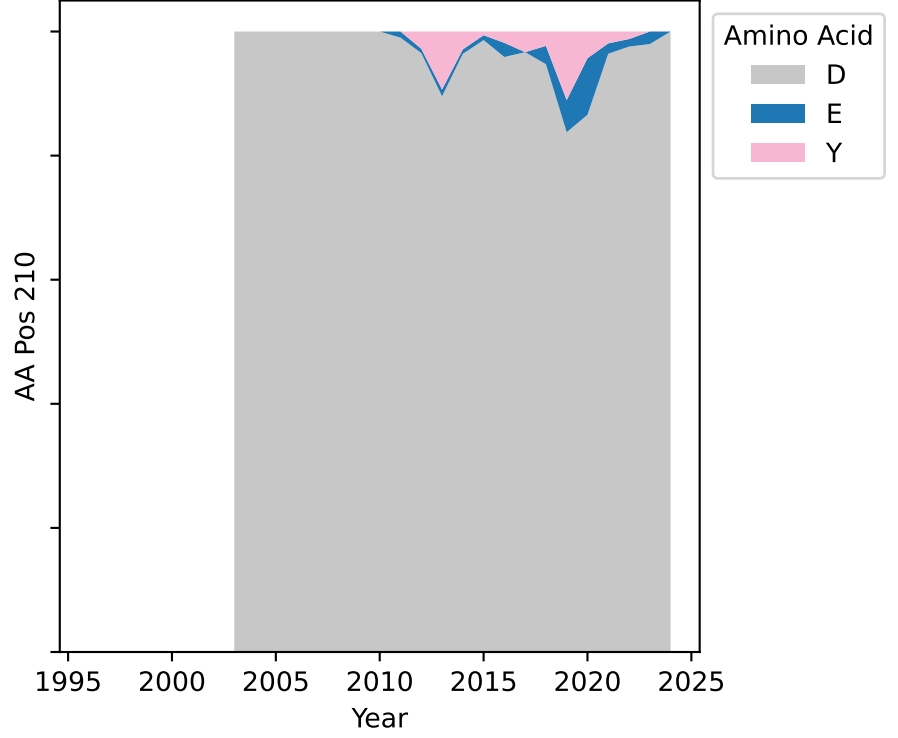

Supplement: Supplementary file 1 [file Data_Sheet_1.ZIP › Supplementary figure 4.pdf]

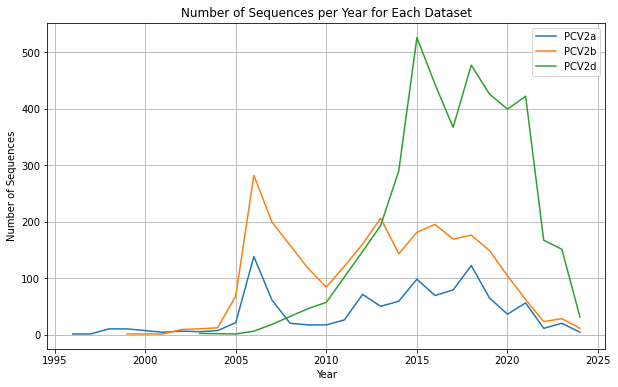

Supplement: Supplementary file 1 [file Data_Sheet_1.ZIP › Supplementary figure 5.png]
