# Supplementary material for: An updated phylogeography and population dynamics of porcine circovirus 2 genotypes: are they reaching an equilibrium?
Source: Front Microbiol. 2024 Oct 29;15:1500498. doi: 10.3389/fmicb.2024.1500498 (PMC11554664; doi:10.3389/fmicb.2024.1500498)
Supplement: Supplementary file 2 [file Data_Sheet_2.DOCX]

Supplementary Material

**
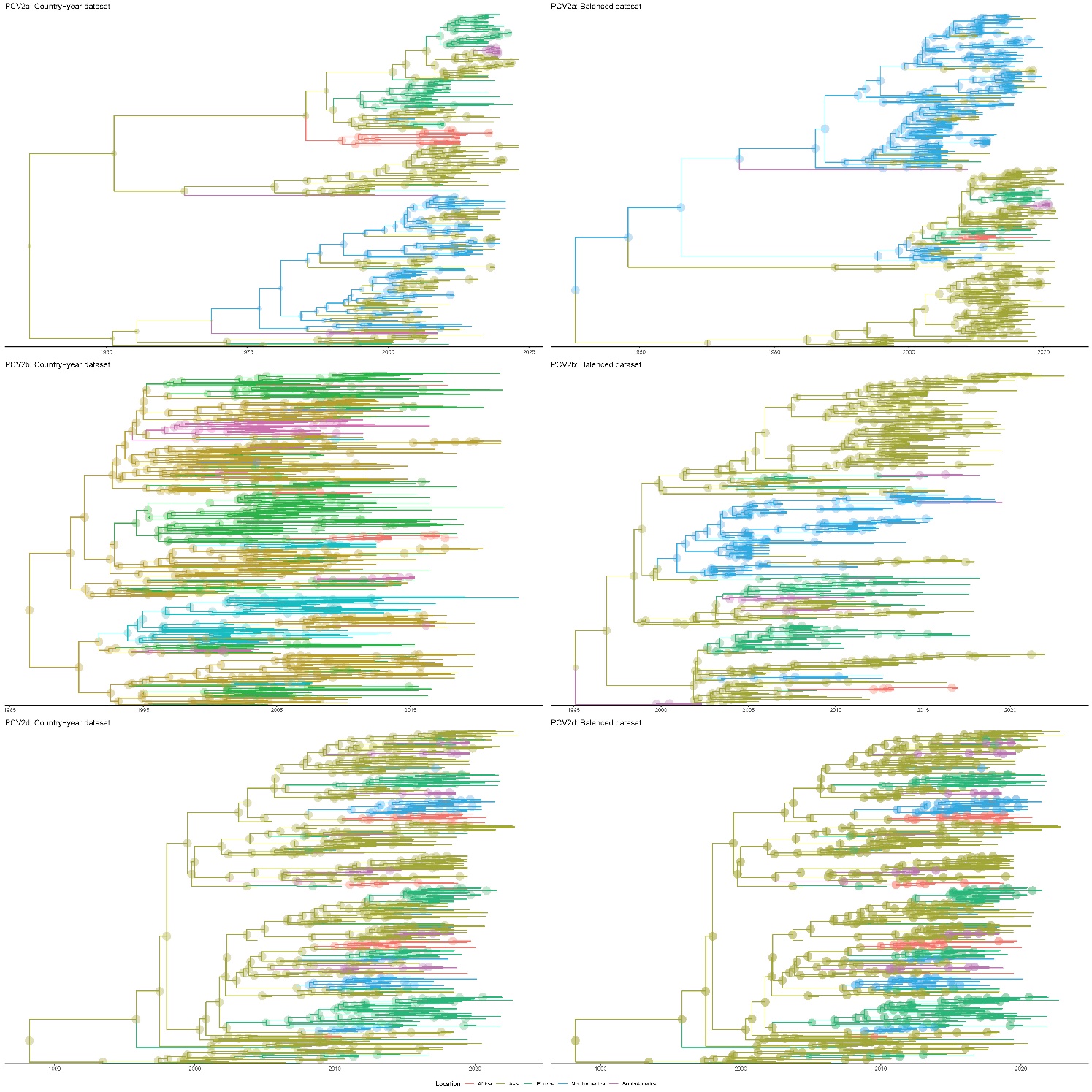
**

**Supplementary Figure 1.** Maximum clade credibility trees based on the PCV2a, PCV2b and PCV2d ORF2 dataset. Tips and branches are color-coded according to the collection area, or the one estimated with the higher posterior probability, respectively. Node size is proportional to the posterior probability of the inferred locations. The results of different datasets are reported in different panels.

**
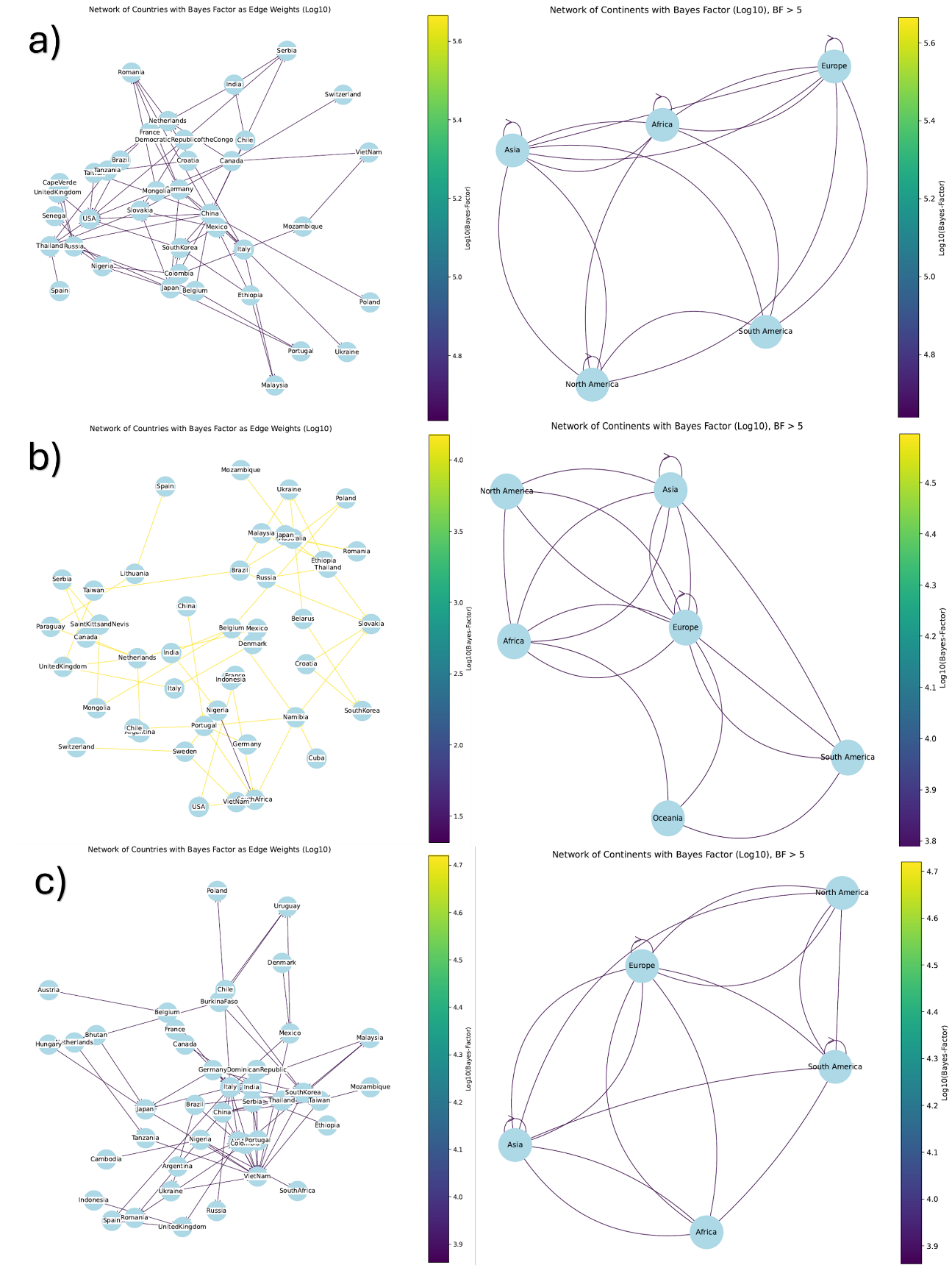
**

**Supplementary Figure 2.** Well-supported migration paths (i.e., Bayesian factor [BF] > 5) of PCV2a (a), PCV2b (b) and PCV2d (c) among countries (left panel) and continents (right panel). The edge color is proportional to the log10 Bayesian factor of the inferred link.

**
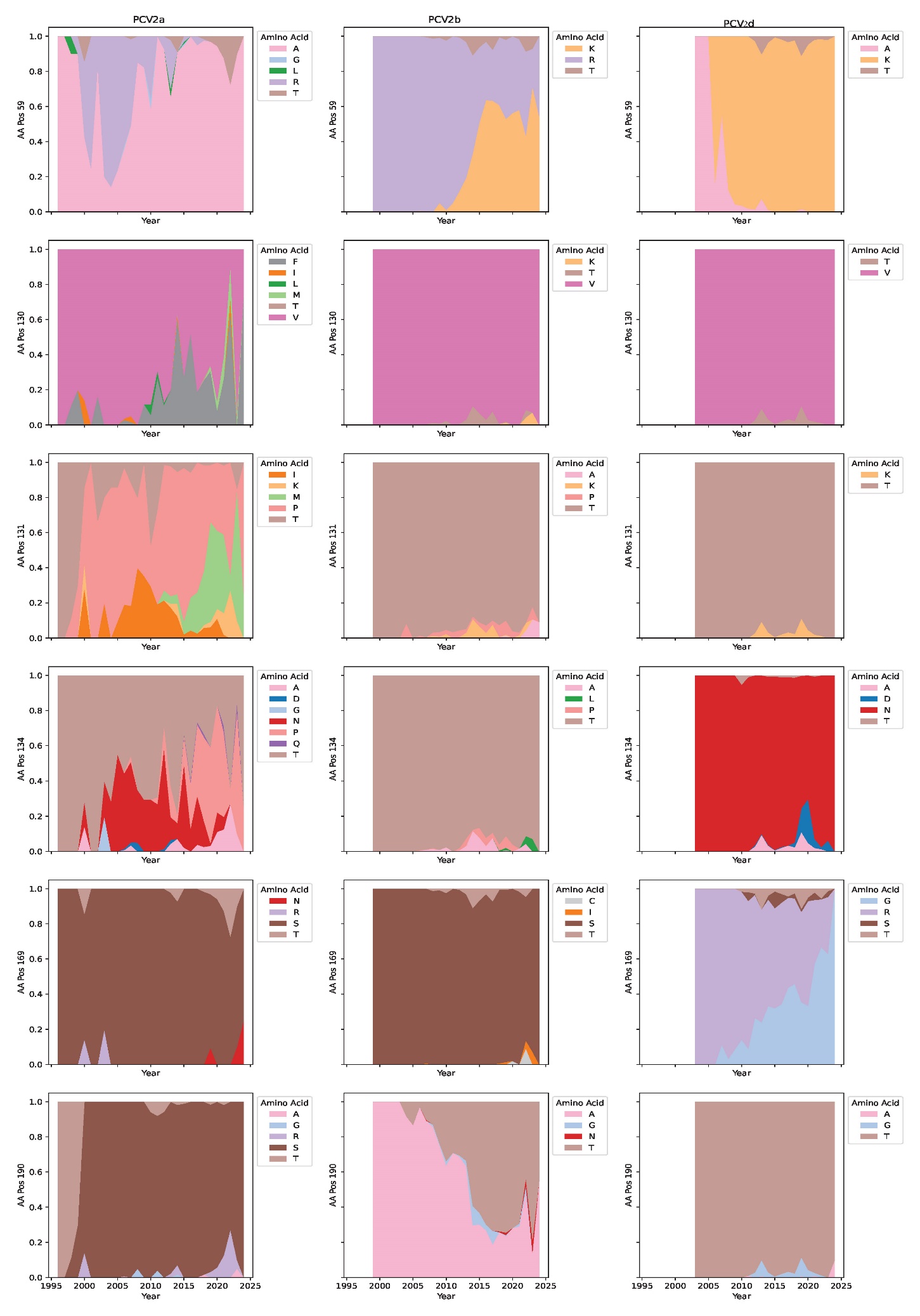
**

**Supplementary Figure 3.** Plot Depicting the Proportion of Amino Acids Over Time at Capsid Positions under Episodic Diversifying selection: This figure illustrates the proportion of amino acids, represented by different colours, in sequences from strains collected at various time points. The amino acid positions are listed as rows, while the PCV2 genotypes are displayed as columns. For graphical clarity, only a subset of amino acids that were detected under significant selective pressure by MEME in at least two genotypes is included in the figure.


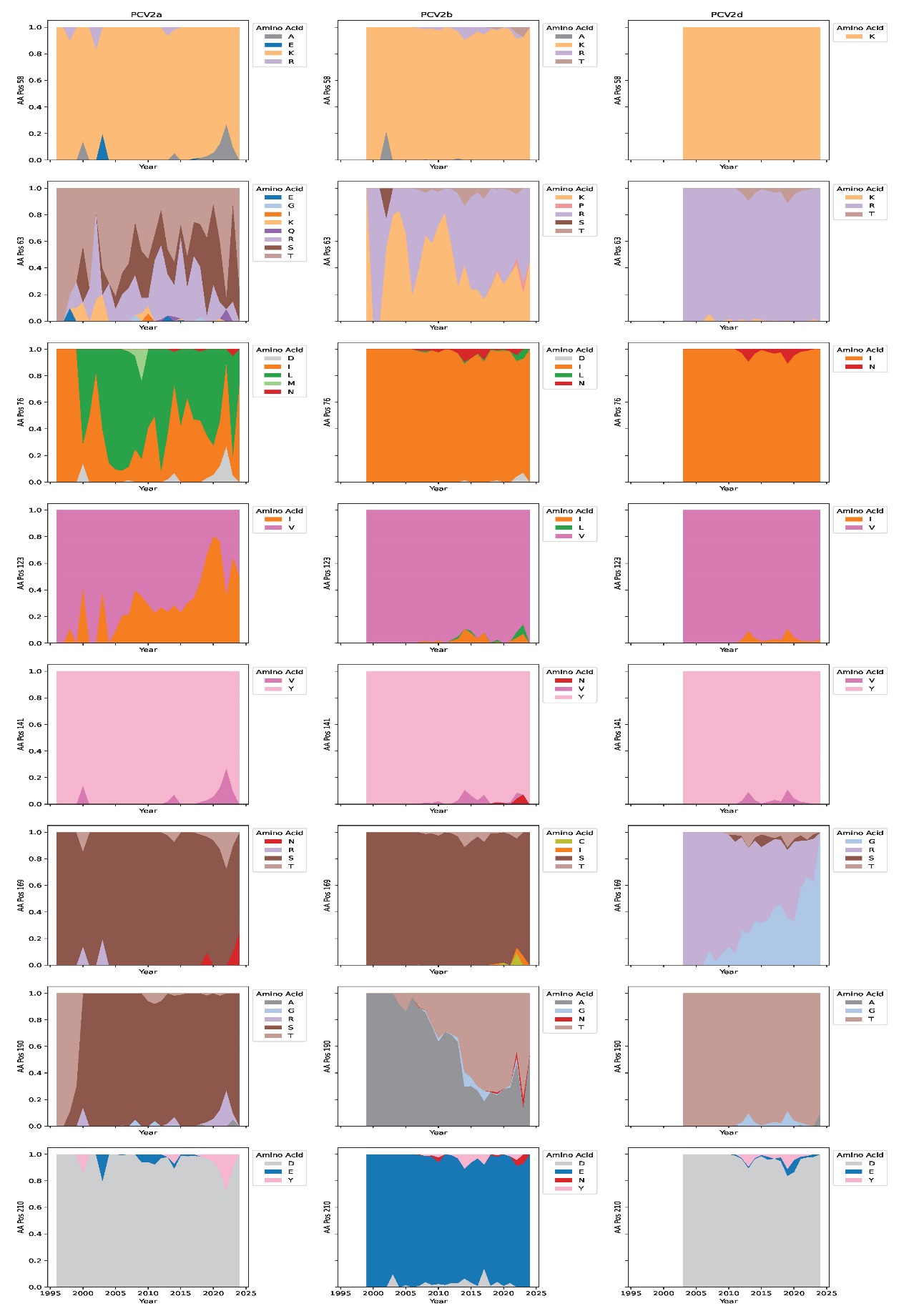


**Supplementary Figure 4.** Plot Depicting the Proportion of Amino Acids Over Time at Capsid Positions under Differential Selective Pressure in Different Genotypes: This figure illustrates the proportion of amino acids, represented by different colours, in sequences from strains collected at various time points. The amino acid positions are listed as rows, while the PCV2 genotypes are displayed as columns. For graphical clarity, only a subset of amino acids that were detected under significant selective pressure by Contrast-FEL in at least two genotypes is included in the figure.


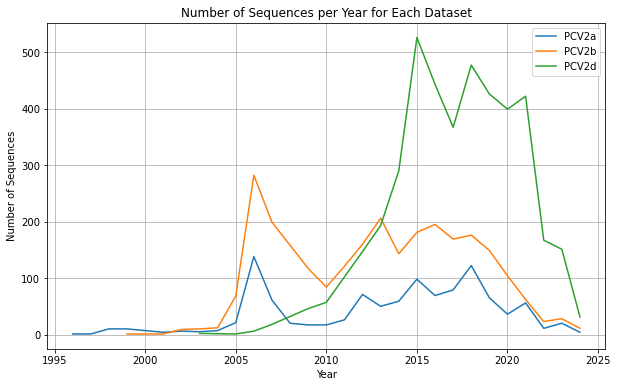


**Supplementary Figure 5.** Plot depicting the count of available sequences of the major PCV2 genotypes based on the strain collection date.

High resolution figures are provided in the Supplementary figure.zip file.
